# Local Distortions in a Prototypical Zeolite Framework Containing Double Four‐Ring Cages: The Role of Framework Composition and Organic Guests**

**DOI:** 10.1002/cphc.202000863

**Published:** 2020-11-27

**Authors:** Michael Fischer, Linus Freymann

**Affiliations:** ^1^ Faculty of Geosciences University of Bremen Klagenfurter Straße 2–4 28359 Bremen Germany; ^2^ MAPEX Center for Materials and Processes University of Bremen 28359 Bremen Germany

**Keywords:** ab initio calculations, host-guest systems, molecular dynamics, zeolites, zeolite analogues

## Abstract

Cube‐like double four‐ring (*d4r*) cages are among the most frequent building units of zeolites and zeotypes. In materials synthesised in fluoride‐containing media, the fluoride anions are preferentially incorporated in these cages. In order to study the impact of framework composition and organic structure‐directing agents (OSDAs) on the possible occurrence of local distortions of fluoride‐containing *d4r* cages, density functional theory (DFT) calculations and DFT‐based molecular dynamics simulations were performed for AST‐type zeotypes, considering four different compositions (SiO_2_, GeO_2_, AlPO_4_, GaPO_4_) and two different OSDA cations (tetramethylammonium [TMA] and quinuclidinium [QNU]). All systems except SiO_2_‐AST show significant deformations, with a pyritohedron‐like distortion of the *d4r* cages occurring in GeO_2_‐ and GaPO_4_‐AST, and a displacement of the fluoride anions towards one of the corners of the cage in AlPO_4_‐ and GaPO_4_‐AST. While the distortions occur at random in TMA‐containing zeotypes, they exhibit a preferential orientation in systems that incorporate QNU cations. In addition to providing detailed understanding of the local structure of a complex host‐guest system on the picosecond timescale, this work indicates the possibility to stabilise ordered distortions through a judicious choice of the OSDA, which might enable a tuning of the material's properties.

## Graphical abstract

**Dynamic ions in small cages**: The equilibrium position and dynamic behaviour of fluoride anions encapsulated in double four‐ring cages are investigated using ab initio molecular dynamics simulations. While the occurrence of local distortions depends on the framework composition, the organic cation determines whether they are randomly oriented or ordered.

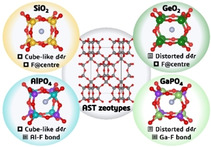

## 1 Introduction

Double four‐ring cages (*d4r* units, face symbol 4^6^, **t‐cub** tile in the nomenclature of natural tilings^[1]^) are a prototypical building unit of zeolites and zeotypes. A recent statistical analysis of the zeolite frameworks included in the IZA (International Zeolite Association) Database of Zeolite Structures^[2]^ showed that the **t‐cub** tile is the second most frequent tile, occurring in 36 out of 239 zeolite frameworks.^[3]^ As *d4r* units consist of four‐membered rings, the T−O−T angles (where T=tetrahedrally coordinated atoms such as Si, Ge, Al, P, …) along the edges are relatively small, typically below 140 degrees. Because the equilibrium Si‐O−Si angle is closer to 150 degrees,[^4^, ^5^] these building units are strained in all‐silica zeolites.^[6]^ The strain can be reduced through an incorporation of heteroatoms, especially germanium, at some corners of the cage,[^4^, ^7^] or through encapsulation of fluoride anions inside the cages.[^8^, ^9^] In fact, many all‐silica zeolites containing *d4r* units have, so far, not been obtained in the absence of fluoride. While the formation of a particular framework type is primarily governed by the organic structure‐directing agents (OSDAs), which are encapsulated in larger cavities, this indicates that the fluoride anions play an important structure‐directing role in the formation of *d4r* cages.^[10]^

Examples of *d4r*‐containing all‐silica zeolites include octadecasil (AST framework type in the IZA nomenclature^[2]^),^[11]^ ITQ‐7 (ISV),^[12]^ ITQ‐12 (ITW),^[13]^ ITQ‐13 (ITH),^[14]^ ITQ‐29 (LTA),^[15]^ HPM‐1 (STW),^[16]^ and IM‐17 (UOV).^[17]^ Some of these neutral‐framework materials have been proposed, for example, for applications in adsorption‐based gas separations (ITQ‐12: propene/propane separation;^[18]^ ITQ‐29: carbon dioxide/methane separation^[19]^) and in hydrogen‐selective membranes,^[20]^ for the storage of mechanical energy through water intrusion/extrusion,[^21^, ^22^] and as low‐*κ* dielectrics.^[23]^ Synthesis in the presence of fluoride is also widely used in the field of germanosilicates, where various extra‐large pore zeolites containing *d4r* units have been reported (“extra‐large pore zeolites” have pore apertures formed by rings of at least 14 T atoms).[^24^, ^25^] The materials IM‐12 (UTL),^[26]^ ITQ‐33 (ITT),^[27]^ ITQ‐37 (‐ISV),^[28]^ ITQ‐44 (IRR),^[29]^ ITQ‐54 (‐IFU),^[30]^ and CIT‐13 (*CTH)^[31]^ fall in this category. Systems containing Ge‐rich *d4r* units have received particular attention due to the possibility to selectively remove Ge from the framework.[^32^, ^33^] Selective Ge removal forms the basis of the so‐called ADOR (assembly‐disassembly‐organisation‐reassembly) mechanism, a versatile strategy to prepare new frameworks that are inaccessible through direct synthesis.[^25^, ^34^] Finally, several phosphate‐based zeotypes with *d4r* cages have been synthesised in fluoride‐containing media, among them the extra‐large pore gallophosphate (GaPO) cloverite (−CLO)^[35]^ and LTA‐type aluminophosphates (AlPOs).[^36^, ^37^] While practical applications of cloverite are severely limited due to its low stability,^[38]^ AlPO_4_‐LTA has been employed in membrane‐based separations^[39]^ and in thermal energy storage.[^40^, ^41^]

X‐ray diffraction investigations of as‐synthesised samples prepared via the “fluoride route” have typically located the fluoride anions at, or close to, the centre of the *d4r* cage in all‐silica zeolites like octadecasil,[^11^, ^42^] ITQ‐7,^[43]^ ITQ‐12,^[13]^ and ITQ‐13,^[14]^ and in GeO_2_ zeotypes like ASU‐7 (ASV),[^44^, ^45^] ASU‐9 (AST),[^44^, ^45^, ^46^] FOS‐5 (BEC),^[47]^ ITQ‐21,^[48]^ and IM‐10 (UOZ).^[49]^ In AlPO_4_ and GaPO_4_ zeotypes, somewhat different locations have been reported for different structures: While the fluoride anions are situated at the centre of the cage in AlPO_4_‐16 (AST)^[50]^ and in cubic LTA‐type AlPO_4_ ^[36]^ and GaPO_4_,^[51]^ off‐centre displacements were observed in rhombohedrally distorted AlPO_4_‐LTA^[37]^ and in the gallophosphate cloverite.^[35]^ This off‐centre displacement, which coincides with a distortion of the *d4r* cages, leads to a certain variation in the distances between fluoride and the Al/Ga atoms at the corners, with Al−F distances in rhombohedral AlPO_4_‐LTA ranging from 2.52 to 2.82 Å, and Ga−F distances from 2.29 to 2.65 Å in cloverite. The propensity of fluoride to form Al−F/Ga−F bonds, often occupying a bridging position between two such atoms, is well known.[^10^, ^52^] Such bonding environments are found, for example, in AlPO_4_ and GaPO_4_ zeotypes with “ring‐opened” *d4r* (*sti* cage) units (SSZ‐51,^[53]^ GaPO_4_‐ZON^[54]^) and in CHA‐ and GIS‐type fluoroaluminophosphates.[^55^, ^56^, ^57^] With typical Al−F distances of ∼1.85 to 2.0 Å, and Ga−F distances of ∼1.9 to 2.2 Å, the distances observed in these zeotypes are shorter than those found in *d4r*‐containing systems,^[10]^ pointing to a different bonding character. The isotropic displacement parameters of F atoms located inside *d4r* cages are often considerably larger than those of the framework atoms.[^37^, ^42^, ^46^] On the one hand, this can be plausibly explained with the considerable mobility of the encapsulated fluoride anions in the absence of a localised bond to any of the surrounding T atoms. On the other hand, it might also point to a disorder over different positions within the cage. If only powder samples are available, as is typically the case for synthetic zeolites and zeotypes, it is challenging to resolve such disorder with diffraction methods.

Whereas diffraction measurements deliver information about the long‐range structure, NMR methods can probe the local environment. ^19^F‐NMR measurements have been widely used to prove the incorporation of fluoride into *d4r* cages, as it is associated with distinct ^19^F chemical shifts.[^10^, ^36^, ^58^] Moreover, NMR can deliver insights into the preferential local arrangements of different elements at the corners of the cage, *e. g*., in mixed (Si,Ge)O_2_ zeotypes.[^33^, ^46^, ^59^, ^60^, ^61^] Electronic structure methods in the framework of density functional theory (DFT) have been employed to study different aspects of fluoride‐containing *d4r* units, such as the nature of the chemical bonding,[^62^, ^63^] characteristic features in vibrational spectra,^[8]^ the mechanism of fluoride removal from the *d4r* cage,^[6]^ and the relationship between different local arrangements of Si and Ge at the corners of the cage and the resulting ^19^F and ^29^Si chemical shifts.[^60^, ^61^, ^64^, ^65^, ^66^] In a previous investigation by one of us, DFT structure optimisations and DFT‐based ab initio molecular dynamics (AIMD) calculations were used to study the local environment and fluoride dynamics in AST‐type germanosilicates.^[67]^ It was found that the fluoride anions reside at the cage centre in the pure end members SiO_2_‐AST and GeO_2_‐AST, but that they are displaced towards one Ge atom, or towards Ge−O−Ge edges, in mixed (Si,Ge)O_2_ systems, often forming directional Ge−F bonds with a length of 2.2 to 2.4 Å. The formation of these bonds leads to a reduction in the freedom of motion that is clearly visible in the root mean square displacements (RMSDs) of fluoride and in the F−Ge radial distribution functions (RDFs). Building on this previous study, the present work uses an analogous methodological approach to investigate several new aspects:

1. As pointed out above, off‐centre displacements have been reported for some, but not all, AlPO_4_ and GaPO_4_ zeotypes containing *d4r* cages, and diffraction methods can provide only limited insights into the preferred local environments. In order to study the dependence of the equilibrium position of fluoride anions, and of their dynamic behaviour, on the composition of the framework, AST‐type zeotypes having four different compositions (SiO_2_, GeO_2_, AlPO_4_, GaPO_4_) are included.
2. The previous computational study dealt exclusively with models containing the highly symmetric tetramethylammonium (TMA) cation as OSDA. However, AST‐type systems like octadecasil^[11]^ and AlPO_4_‐16[^50^, ^68^] can also be synthesised using the less symmetric quinuclidinium (QNU) cation. Given its very different molecular structure, notably the presence of a terminal −NH group, different framework‐OSDA interactions can be anticipated, especially in terms of hydrogen bonding. To this end, TMA‐ and QNU‐containing models are compared to investigate if and how the organic cation affects the equilibrium position and dynamics of the fluoride anions.
3. Previous work on TMA‐containing octadecasil postulated the existence of weak C−H⋅⋅⋅O “hydrogen bonds” between methyl groups and framework oxygen atoms.[^62^, ^69^] The computations performed in this study can give further insights into the potential existence of these bonds, and their significance at finite temperatures.
4. In order to investigate the effect of temperature, AIMD simulations were performed for three temperatures (150 K, 298 K, 573 K), whereas previous work exclusively studied the behaviour at 298 K (room temperature).

The central motivation of the present work is the development of a more detailed atomic‐level picture of the local structure of these systems, as the insights obtainable with commonly used experimental methods are limited. While this is primarily of fundamental interest, the findings could eventually contribute to an improved understanding of the formation of zeolites and zeotypes during fluoride‐mediated synthesis, which might aid the development of new or improved synthesis strategies. Furthermore, the presence of local distortions could also affect potential applications of these materials, especially those related to the dielectric properties.

It has to be noted that AST‐type zeolites and zeotypes are of little relevance for applications because their pores are inaccessible to most guest molecules. However, they constitute an ideal model system in the present context for the following reasons: First, AST‐type materials have been synthesised in SiO_2_, GeO_2_, and AlPO_4_ composition, so only GaPO_4_‐AST is a purely hypothetical system. Second, prior experimental studies have shown that each of the larger octadecahedral (*ast*) cages is occupied by a single OSDA molecule, rather than several molecules. This facilitates the construction of models for the calculations. Given the similarity of the local environment, it can be expected that the findings obtained for AST in the present work are, to a large degree, transferable to other zeolites/zeotypes with *d4r* cages (probably excepting those where the cages are strongly distorted).

## 2 Models and Methods

### 2.1 Structure Models

**Figure 1:**
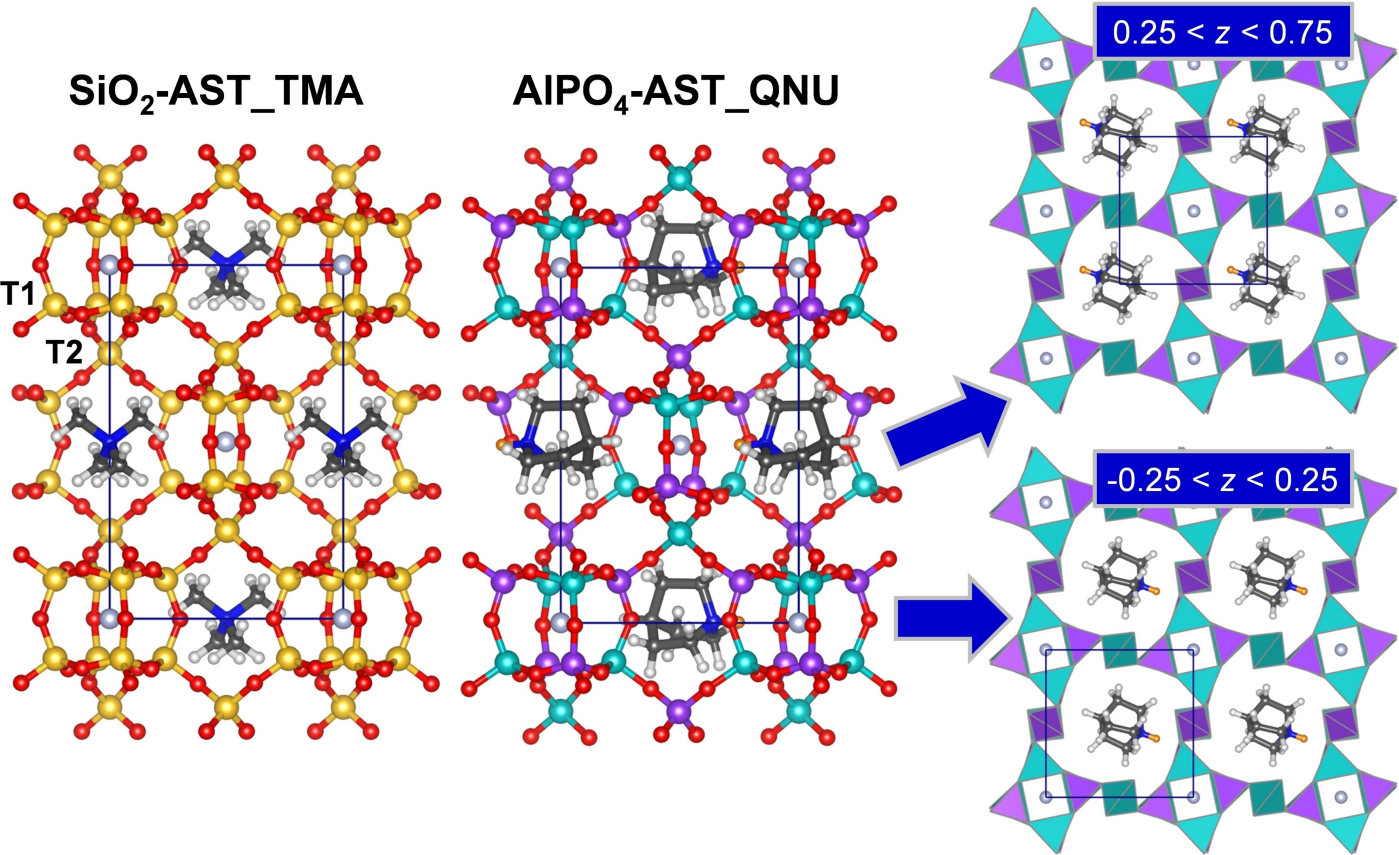
**Left**: Unit cell of SiO_2_‐AST_TMA. **Middle**: Unit cell of AlPO_4_‐AST_QNU. **Right**: Visualisation of AlPO_4_‐AST_QNU indicating the different orientations of QNU molecules in planes perpendicular to *c*. Colour scheme: red=O, yellow=Si, cyan=Al, purple=P, light blue=F, dark blue=N, dark grey=C, white=H. The hydrogen atom bonded to the QNU nitrogen atom is highlighted in orange.

Throughout this work, labels including both framework composition and OSDA are used to distinguish different systems (*e. g*., SiO_2_‐AST_TMA=all‐silica AST incorporating TMA cations; as fluoride anions are present in all models, they are not included in the label). The starting models of the framework structure of SiO_2_‐, GeO_2_‐, and AlPO_4_‐AST were taken from experimental crystal structure data.[^11^, ^42^, ^46^, ^50^] Since GaPO_4_‐AST has, to the authors’ knowledge, not yet been synthesised, this model was constructed on the basis of AlPO_4_‐AST. In all starting structures, the fluoride anions were located at the centre of the *d4r* cages. The cationic OSDAs in the larger *ast* cages are disordered in the experimental structures. As this disorder had to be removed prior to the DFT calculations, some assumptions regarding the orientation of OSDAs in adjacent cages with respect to each other were necessary. The TMA cation has no dipole moment, so it seems plausible to assume that the orientation of a cation in one cage does not have a strong influence on cations in other cages. Therefore, it was assumed that all TMA molecules have the same orientation, preserving the body‐centering of the structure and leading to $I\bar{4}$ symmetry (Figure 1).^[67]^ The situation is different for the QNU cation, which has a dipole moment. An essentially random orientation of the QNU molecules would require the use of a large supercell, and an identical orientation of all molecules (which would preserve the body‐centering) would lead to an overall polarisation, which appears unlikely. In order to avoid both issues, an arrangement was used in which all QNU molecules in one plane perpendicular to the *c* axis (around *z*=0) point in one direction, whereas those in the other plane (around *z*=0.5) point in the opposite direction (Figure 1).

### 2.2 Computational Details

DFT structure optimisations and DFT‐based AIMD simulations were performed using the quickstep part^[70]^ of the CP2K code,^[71]^ installed on the HLRN‐III/HLRN‐IV facilities of the North‐German Supercomputing Alliance. All calculations employed the PBE exchange‐correlation functional^[72]^ in conjunction with Grimme's D3 dispersion correction.^[73]^ This PBE‐D3 functional delivered fairly accurate results in a recent benchmarking study of zeolites and zeotypes,^[74]^ and it was successfully used in previous AIMD investigations of fluoride‐containing zeolites.[^67^, ^75^] A plane‐wave energy cutoff of 600 Ry was used, and the first Brillouin zone was sampled at the Γ point, only, in keeping with previous work.^[67]^ The calculations employed Goedeker‐Teter‐Hutter pseudopotentials devised by Krack.^[76]^ Gaussian triple‐zeta (TZVP/TZVP‐SR) basis sets were used in the structure optimisations, whereas the AIMD simulations used double‐zeta (DZVP‐SR) basis sets.^[77]^ These basis sets are available in the basis_molopt_ucl (TZVP‐SR basis sets for Al, Ga, Ge) and basis_molopt (all others) files distributed with the CP2K code.

All structure optimisations were carried out using the tetragonal unit cell of AST‐type zeolites (*a*≈9 Å, *c*≈14 Å). The atomic positions and unit cell parameters were optimised, fixing the unit cell to a tetragonal metric (*a*=*b*, *α*=*β*=*γ*=90°), and using the following convergence criteria: maximal geometry change (step size)=2×10^−5^ bohr, maximal residual force (gradient)=10^−6^ Ha bohr^−1^, and maximal pressure deviation=0.01 GPa. The AIMD simulations, which started from the optimised structures, employed a 2×2×1 supercell. These simulations were performed in the canonical (*NVT*) ensemble for temperatures of 150 K, 298 K, and 573 K, using a Nosé‐Hoover thermostat[^78^, ^79^] with a timestep of 0.5 fs and a time constant of 50 fs. Three independent AIMD simulations covering 10 ps (20,000 steps) were run for each system at each temperature. The first 2.5 ps (5,000 steps) from each trajectory were discarded (equilibration phase), and the remaining 7.5 ps (15,000 steps) were used in the analysis (production phase). The analysis made use of the VMD code, version 1.9.3,^[80]^ to calculate radial distribution functions (RDFs) for selected combinations of elements, root mean square displacements (RMSDs), and average structures (=average atomic positions over the whole 7.5 ps). All RDFs and RMSDs discussed throughout this work correspond to averages over three independent trajectories. The numerical results are compiled in an EXCEL file that has been deposited in the Figshare repository under https://doi.org/10.6084/m9.figshare.12981557.v1. Archives containing the trajectories in PDB format (production phase only), DFT‐optimised structures and AIMD average structures in CIF format, and sample input files have been deposited in the same repository. All structure visualisations in this article were prepared using vesta.^[81]^

## 3 Results and Discussion

The first part of this section presents the results of the DFT optimisations, with most emphasis on the local structure of the *d4r* units. The second part addresses the AIMD simulation results. After giving a brief overview of the RMSDs of framework atoms and fluoride anions, the local environments of fluoride anions are investigated in more detail by analysing RDFs and AIMD average structures. As the behaviour differs considerably as a function of framework composition, these results are presented separately for each zeotype. The role of OSDA dynamics and framework‐OSDA hydrogen bonds is discussed in the last part.

### 3.1 DFT Structure Optimisations

Prior to discussing the optimisation results, a brief description of the AST framework is warranted. In the cubic aristotype (space group $Fm\bar{3}m$ ), the T sites at the corners of the *d4r* cages, labelled T1, are equivalent. The *d4r* cages are connected to each other via linkages to another type of T atom, labelled T2 (Figure 1). While the edges of the *d4r* cages are formed by T1−O−T1 linkages, T1−O−T1 and T1−O−T2 linkages together form the edges of larger *ast* (octaedecasil) cages (face symbol 4^6^ ⋅ 6^12^). The T1−O−T2 angle in the cubic aristotype is fixed to 180° for symmetry reasons.^[68]^ Due to the instability of such straight linkages,^[82]^ a reduction in symmetry occurs in real AST‐type materials: Whereas materials with T=Si and Ge possess I4/m symmetry,[^11^, ^42^, ^83^] those with two different species at the T sites, like AlPO_4_‐16, crystallise in space group $I\bar{4}$ .[^50^, ^84^] When performing DFT calculations, a further reduction in symmetry is often necessary because the disorder of the OSDA molecules needs to be removed, as discussed above.

The unit cell parameters of the DFT‐optimised AST systems are summarised in Table 1. When comparing pairs of systems having the same framework composition, but containing different OSDAs, it is worth noting that the incorporation of QNU leads to an expansion of the *a* axis and a concurrent contraction of the *c* axis when compared to the corresponding TMA‐containing system. Clearly, this is related to the elongated shape of the QNU molecules, which are oriented in a way that the longest molecular dimension lies in the *ab* plane. Among systems containing the same OSDA, but having different framework composition, replacing elements from the 3^rd^ period (Si, Al) by elements from the 4^th^ period leads to an expansion of the unit cell, especially along the *c* axis, in line with the increase of the atomic radius of the T atoms. Experimental cell parameters are available for three of the eight systems, and the corresponding values are listed in Table 1. Compared to the experimental values, the DFT calculations deliver a shorter *a* axis and a longer *c* axis. Similar tendencies were observed and discussed in previous DFT studies of calcined SiO_2_‐AST.[^85^, ^86^] The effect is fairly prominent for AlPO_4_‐AST_QNU, where the relative deviation in *a* exceeds −2 %, and moderately pronounced for SiO_2_‐AST_TMA. In contrast, agreement with experiment is excellent for SiO_2_‐AST_QNU.

**Table 1:** Results of DFT optimisations: Unit cell parameters and comparison to experimental data (where available), space group resulting after symmetry search for the complete system (Fw+OSDA) and for the bare framework after removal of OSDA cations and fluoride anions (Fw).

			*a*/[Å]	*c*/[Å]	*Space group Fw+OSDA*	*Space group Fw*
SiO_2_‐AST	TMA	DFT	8.970	13.605	$I\bar{4}$	I4/m
		Exp^[42]^	9.068	13.438	I4/m	
	QNU	DFT	9.214	13.466	$P2_{1}$	I4/m
		Exp^[11]^	9.194	13.396	I4/m	
GeO_2_‐AST	TMA	DFT	9.151	14.635	$I\bar{4}$	I4/m
	QNU	DFT	9.476	14.101	$P2_{1}$	I2/m
AlPO_4_‐AST	TMA	DFT	8.982	13.772	$I\bar{4}$	$I\bar{4}$
	QNU	DFT	9.145	13.657	$P2_{1}$	$I\bar{4}$
		Exp^[50]^	9.342	13.476	$I\bar{4}$	
GaPO_4_‐AST	TMA	DFT	8.971	14.132	$I\bar{4}$	$I\bar{4}$
	QNU	DFT	9.191	13.963	$P2_{1}$	$I\bar{4}$

As the CP2K optimisations do not make use of symmetry information, slight deviations from ideal symmetry occur in the DFT‐optimised structures (up to a few 1/100 Å). In order to restore the symmetry prior to the analysis, a symmetry search was carried out using Materials Studio.^[87]^ The results, tabulated in Table 1, show that all TMA‐containing systems have $I\bar{4}$ symmetry, the highest possible symmetry permitted by the chosen arrangement of the OSDA molecules in the *ast* cages.^[67]^ All QNU‐containing systems have symmetry $P2_{1}$ after the symmetry search. The reduction in symmetry is related to a) the lower symmetry of the OSDA, which removes the fourfold rotoinversion axis, and b) the relative orientation of the QNU molecules in planes perpendicular to *c*, visualised in Figure 1, which removes the body‐centering.

**Figure 2:**
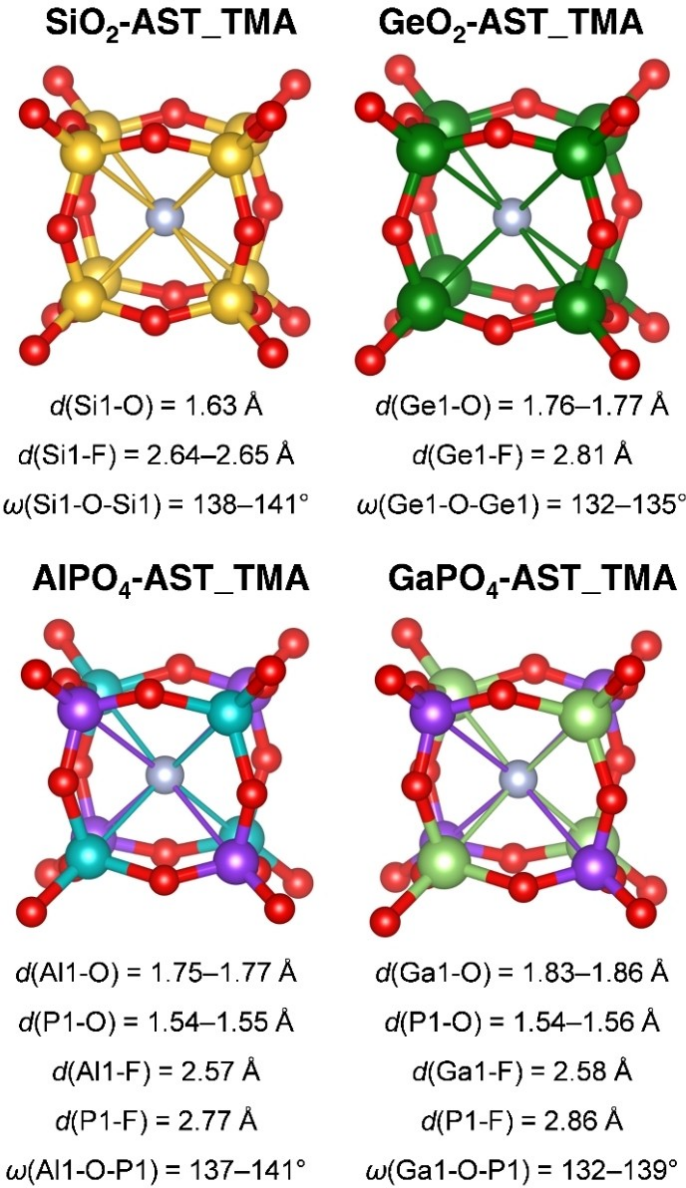
*d4r* cages in DFT‐optimised structures of AST_TMA zeotypes. Colour scheme: dark green=Ge, light green=Ga, see caption of Figure 1 for other colours. Thin lines between T atoms and fluoride anions are included to emphasise the location of fluoride close to the cage centre.

The *d4r* cages of the DFT‐optimised AST_TMA systems are shown in Figure 2, which also reports the ranges of T−O and T−F distances and T−O‐T angles along the cage edges (*i. e*., T=T1). The T−O distances fall into the respective ranges determined in a statistical analysis of experimental crystal structures,^[88]^ they are not further discussed here. In SiO_2_‐ and GeO_2_‐AST_TMA, the fluoride anions are located at the cage centre, as evidenced by a very narrow distribution of the T−F distances. The distances agree well with those determined experimentally for AST‐type SiO_2_ and GeO_2_ systems (*d*(Si−F)_exp_=2.63 Å, *d*(Ge−F)_exp_=2.75 Å).[^42^, ^46^] The Ge−O−Ge angles are significantly smaller than the Si−O−Si angles, in line with the smaller equilibrium angle.[^4^, ^5^, ^45^, ^89^, ^90^] In AlPO_4_‐ and GaPO_4_‐AST_TMA, the distributions of Al/Ga−F and P−F distances are also very narrow, with the Al/Ga−F distances being systematically shorter than the P−F distances. The same trend has been observed in crystallographic studies of *d4r*‐containing alumino‐ and gallophosphates,[^35^, ^36^, ^50^, ^51^] and quantitative agreement with experimental T−F distances is satisfactory. An inspection of O−T−O angles (for O atoms occupying edges of the *d4r* cage) shows larger deviations from the ideal tetrahedral angle for AlO_4_/GaO_4_ tetrahedra compared to PO_4_ tetrahedra, with individual O−Al−O/O−Ga−O angles reaching 118°/121°, respectively (O−P‐O angles do not exceed 114°). Apparently, the AlO_4_/GaO_4_ tetrahedra are less rigid than the PO_4_ tetrahedra, and attractive electrostatic interactions with the fluoride anions cause a certain displacement of the metal cations into the cage. The higher degree of flexibility can be explained with the more ionic (= less directional) nature of the Al−O/Ga−O bonds in comparison to the dominantly covalent P−O bonds.^[91]^

A visualisation of the TMA cations and the surrounding *ast* cages is provided in the Supporting Information, Figure S1. This figure shows that two or three hydrogen atoms of each methyl group form relatively close (<2.7 Å) contacts to framework oxygen atoms, with H⋅⋅⋅O distances of 2.57/2.66 Å in SiO_2_‐AST_TMA, 2.44/2.52 Å in GeO_2_‐AST_TMA, 2.68/2.64 Å in AlPO_4_‐AST_TMA, and 2.63/2.51/2.60 Å in GaPO_4_‐AST_TMA. These distances appear to be too long to serve as unambiguous evidence for the presence of C−H⋅⋅⋅O hydrogen bonds, which have been postulated in a previous NMR study of SiO_2_‐AST_TMA.^[69]^ On the other hand, they are up to ∼10 % smaller than the sum of van der Waals radii of hydrogen and oxygen of 2.70 Å,^[92]^ indicating a non‐negligible electrostatic interaction.

**Figure 3:**
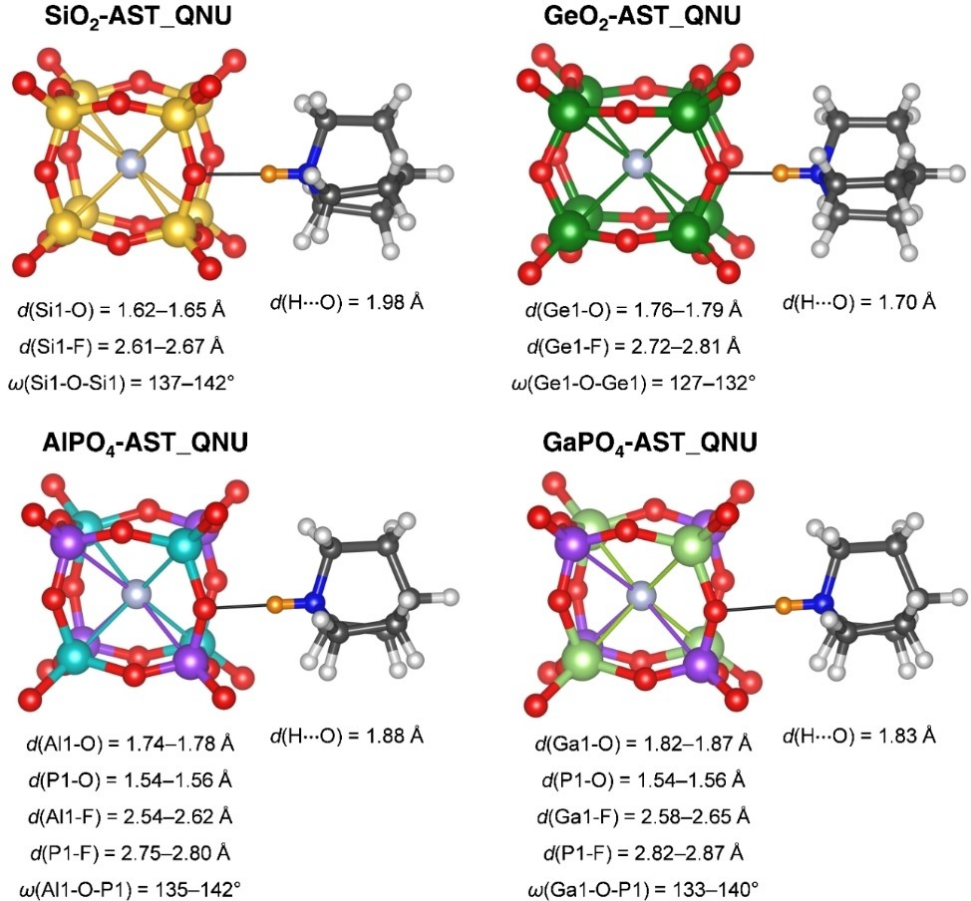
*d4r* cages and adjacent QNU cations in DFT‐optimised structures of AST_QNU zeotypes. Thin lines between T atoms and fluoride anions are included to emphasise the location of fluoride close to the cage centre.

Figure 3 shows one *d4r* cage of the DFT‐optimised AST_QNU structures as well as the adjacent QNU cation that is hydrogen‐bonded to a framework oxygen atom via the −NH group (each *d4r* cage is surrounded by six QNU cations, but only one of them is hydrogen‐bonded to an O atom belonging to the cage). In all four cases, the T−F distances show a slightly broader distribution than in the AST_TMA systems. When looking at the individual distances, it becomes apparent that the fluoride anions are slightly displaced from the cage centre towards the hydrogen‐bonded QNU cation; however, this displacement amounts only to a few 1/100 Å. The T−O−T angles along the edges are rather similar to those in AST_TMA systems, with the exception of GeO_2_‐AST_QNU, where they are systematically smaller. This reduction in angles coincides with a pronounced distortion of the *d4r* cage. While the Ge−Ge distances are hardly affected, the O−O distances measured across the faces show a bimodal distribution, with “short” O−O distances of about 3.4 Å and “long” O−O distances of about 4.7 Å (Figure S3). In contrast, all O−O distances in GeO_2_‐AST_TMA fall close to 4.2 Å. Similarly distorted *d4r* cages have been previously observed in a crystallographic study of the germanate ASU‐7, where they were described as rectangular prisms.^[93]^ A closer look at these units reveals that the idealised symmetry is higher than that of a general rectangular prism (point group mmm ), because three faces meeting at each corner are related by a threefold rotation axis (point group $m\bar{3}$ ). This feature can be illustrated by drawing auxiliary O−O connections across the faces. As shown in Figure S3, the resulting shape resembles a pyritohedron, *i. e*., an irregular pentagonal dodecahedron consisting of identical, but irregular pentagons. In the following, these distorted *d4r* cages will be referred to as “pyritohedron‐like” in order to distinguish them from undistorted “cube‐like” cages. It is worth noting that published crystal structures of the GeO_2_ zeotypes ASU‐7 and FOS‐5 contain *d4r* units whose geometry deviates only slightly from point group symmetry $m\bar{3}$ .[^47^, ^93^]

Prior to a further analysis of the framework distortion in GeO_2_‐AST_QNU, it is useful to take a look at the N−H⋅⋅⋅O hydrogen bonds. The H⋅⋅⋅O distances are included in Figure 3. This distance is longest in SiO_2_‐AST_QNU, with 1.98 Å, and shortest in GeO_2_‐AST_QNU, with 1.70 Å, indicating a pronounced difference in the hydrogen bond strength. The relatively short, strong hydrogen bond in GeO_2_‐AST_QNU can be explained with the less obtuse Ge−O−Ge angles, which render the oxygen atom more exposed, resulting in an increased hydrogen bond acceptor ability. Besides, electron density maps show a significantly higher valence electron density along the H⋅⋅⋅O connection in comparison to the other QNU‐containing zeotypes, pointing to a more covalent character of the hydrogen bond (Figure S4).

The symmetry search was repeated for models in which the extra‐framework content (fluoride anions, OSDA cations) was removed (last column of Table 1). Removal of the extra‐framework species results in space group I4/m for SiO_2_‐/GeO_2_‐AST models with the exception of GeO_2_‐AST_QNU, and space group $I\bar{4}\;$ for AlPO_4_‐ and GaPO_4_‐AST. The reduction in symmetry of GeO_2_‐AST_QNU to I2/m is related to the presence of the pyritohedron‐like *d4r* cages, which have lost the fourfold rotation symmetry. A comparison of the two GeO_2_‐AST frameworks (Figure S5) reveals that the GeO_4_ tetrahedra in GeO_2_‐AST_QNU are rotated with respect to their orientation in the TMA‐containing system. The rotation of tetrahedra occurs at both the T1 and the T2 sites, and results in the distortion of the *d4r* cages observed above. It is interesting to note that the T2−O−T1 angles increase as a consequence of these concerted rotations, from ∼127° in GeO_2_‐AST_TMA to ∼131° in GeO_2_‐AST_QNU (Figure S1/S2).

Taken together, there are two possible explanations for the framework distortion observed in GeO_2_‐AST_QNU, and its absence in GeO_2_‐AST_TMA:

1. The distorted framework (I2/m ) is intrinsically more stable, possibly due to the narrower distribution of Ge−O−Ge angles. The DFT optimisation of GeO_2_‐AST_TMA found only a local minimum, but not a global minimum, due to the high symmetry of the starting structure.
2. The stabilisation of the I2/m structure over the I4/m structure is related to framework‐OSDA interactions, specifically, the formation of hydrogen bonds between QNU molecules and framework oxygen atoms.

This point will be revisited when discussing the MD results for the two GeO_2_‐AST systems.

### 3.2 DFT‐Based Molecular Dynamics Simulations

#### 3.2.1 RMSDs of Framework Atoms and Fluoride Anions

Prior to discussing radial distribution functions and AIMD average structures for the individual systems, it is useful to take a look at the RMSDs computed for framework atoms and fluoride anions (large standard deviations and significant scatter severely restrict the possibilities of a meaningful analysis of the OSDA atoms’ RMSDs). They are tabulated in the Supporting Information (Table S1). Altogether, the RMSDs of framework T and O atoms have similar values at 150 K for all eight systems (RMSD(T)≈0.11 Å, RMSD(O) ≈0.16 Å), but they show a more pronounced increase with temperature for GeO_2_‐AST, AlPO_4_‐AST, and, most markedly, GaPO_4_‐AST when compared to SiO_2_‐AST. With regard to the fluoride anions, the F‐RMSDs at 150 K are similar to those of the framework oxygen atoms in SiO_2_‐/AlPO_4_‐AST, but significantly larger in GeO_2_‐/GaPO_4_‐AST. The larger freedom of motion can be attributed to the larger dimensions of the *d4r* cages, a consequence of the longer Ge−O/Ga−O bonds. The increase of the F‐RMSDs with temperature is more pronounced in the zeotypes containing lighter T atoms, resulting in fairly similar F‐RMSDs at 573 K in all systems (between ∼0.37 and ∼0.45 Å with a typical standard deviation of 0.05 Å). While some observations point to qualitative differences in the dynamic behaviour among the different zeotypes considered, especially with regard to the fluoride anions, the findings remain rather tentative. As demonstrated below, the analysis of RDFs and average structures can provide more insights in this regard.

#### 3.2.2 Fluoride Dynamics and Framework Distortions

##### 3.2.2.1 SiO_2_‐AST

**Figure 4:**
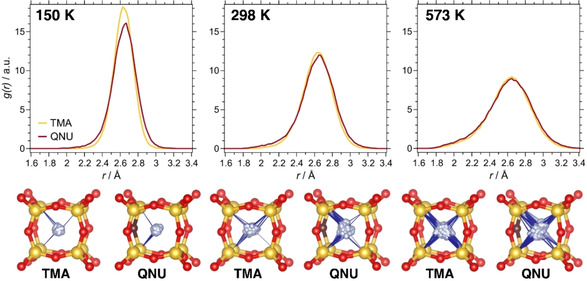
**Top**: F−Si RDFs in SiO_2_‐AST_TMA and SiO_2_‐AST_QNU. **Bottom**: Trajectories of individual fluoride anions. The positions of Si and O atoms are taken from the respective average structures. Blue lines are used to indicate Si−F contacts below 2.3 Å. The oxygen atom that acts as hydrogen bond acceptor is shown in brown.

The F−Si RDFs of SiO_2_‐AST systems are shown in Figure 4 and the average structures computed from individual trajectories are visualised in Figures S6a and S7a. The F−Si RDFs of SiO_2_‐AST_TMA show a symmetric maximum centred at ∼2.65 Å, which corresponds to oscillations of fluoride about its equilibrium location at the centre of the cage, replicating the findings from the earlier AIMD study of this system.^[67]^ Due to increased thermal motion, the maximum becomes broader with increasing temperature. The lower part of Figure 4 overlays the trajectories of one individual fluoride anion inside a *d4r* cage with the time‐averaged positions of the surrounding framework atoms. It is apparent that the number of short Si−F contacts (below 2.3 Å) increases with temperature due to the more vigorous motion of the fluoride anion, and that such short contacts occur without any preferential direction of displacement.

Compared to the TMA‐containing system, the F−Si RDFs of SiO_2_‐AST_QNU show some rather intricate differences, with more prominent “shoulders” at both smaller and larger F−Si distances that are best visible at 150 K. This observation can be attributed to a certain off‐centre displacement of fluoride. The slight change in the equilibrium location is hardly visible in the average structures (Figure S7a), but it becomes more apparent when looking at the trajectory of an individual fluoride anion (lower part of Figure 4): At a temperature of 150 K, the fluoride anion is preferentially displaced towards the Si−O−Si linkage whose oxygen atom participates in the hydrogen bond. With increasing temperature, this effect becomes less pronounced due to the increased overall motion.

##### 3.2.2.2 GeO_2_‐AST

**Figure 5:**
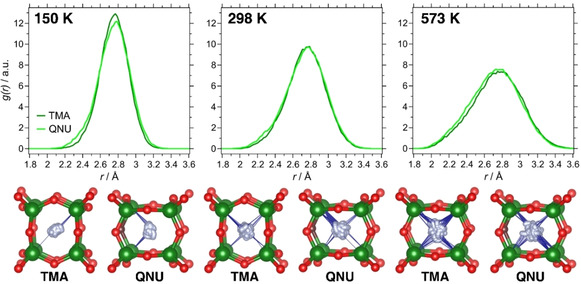
**Top**: F−Ge RDFs in GeO_2_‐AST_TMA and GeO_2_‐AST_QNU. **Bottom**: Trajectories of individual fluoride anions. The positions of Ge and O atoms are taken from the respective average structures. Blue lines are used to indicate Ge−F contacts below 2.3 Å.

**Figure 6:**
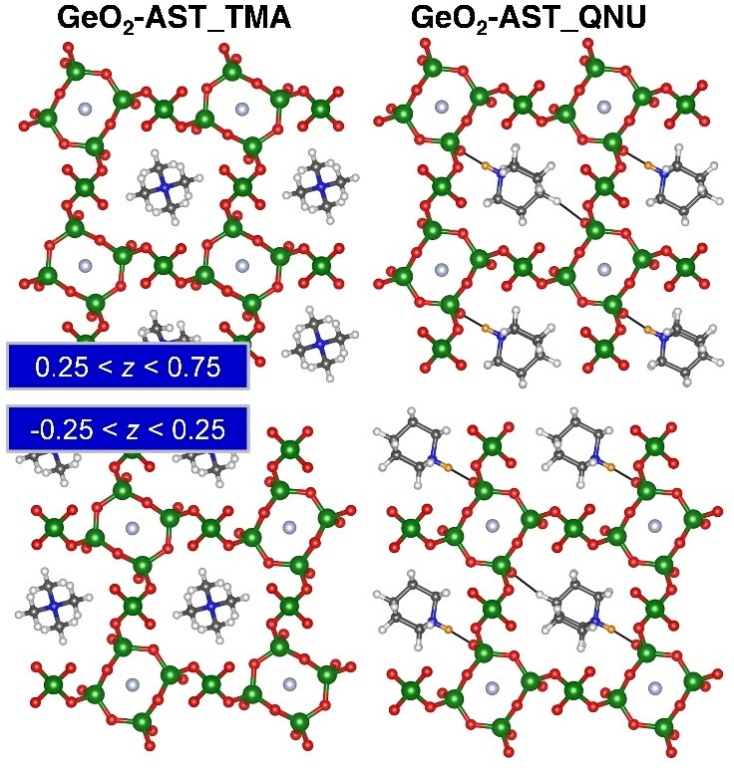
Representative average structures of GeO_2_‐AST_TMA and GeO_2_‐AST_QNU at 150 K. For clarity, different sections perpendicular to *c* are visualised separately.

The F−Ge RDFs as well as trajectories of individual fluoride anions are visualised in Figure 5. Altogether, the findings mirror those discussed above for SiO_2_‐AST systems: The RDFs show a single maximum, centred at a distance of about 2.75 Å. In GeO_2_‐AST_TMA, the fluoride anions oscillate about the cage centre, whereas they are slightly displaced towards the oxygen atom participating in the hydrogen bond in GeO_2_‐AST_QNU. Again, this displacement is best visible at 150 K. Neither system delivers any indications for the presence of Ge−F bonds that have been found to occur in mixed (Si,Ge) *d4r* cages.^[67]^ More interesting observations can be made when looking at the shape of the *d4r* cages. As discussed above, the DFT optimisations deliver a highly symmetric (cube‐like) cage in GeO_2_‐AST_TMA, but a distorted pyritohedron‐like *d4r* cage in GeO_2_‐AST_QNU. An inspection of the average structures of GeO_2_‐AST_TMA (Figure S8a), for which a representative example is shown in Figure 6, reveals that all *d4r* cages exhibit this pyritohedron‐like distortion, causing the loss of tetragonal symmetry described above. This indicates that GeO_2_‐AST containing pyritohedron‐like cages is intrinsically more stable than the higher‐symmetry form with cube‐like *d4r* cages, and that the distortion is not governed by the OSDA. A re‐optimisation of a selected 150 K average structure and the $I\bar{4}$ structure, using the same supercell, confirms this, as the structure with pyritohedron‐like *d4r* cages is favoured by −2.5 kJ mol^−1^ per GeO_2_ formula unit (−26 kJ mol^−1^ per fluoride anion).

Due to the reduced symmetry, two different orientations are possible for the pyritohedron‐like *d4r* cages. In GeO_2_‐AST_TMA, both orientations occur randomly, and such a statistical distribution has been dubbed “orientational glass” in previous studies of AST‐ and ASV‐type systems.[^45^, ^93^] In contrast, a strict ordering is observed in GeO_2_‐AST_QNU at 150 and 298 K, which can be attributed to the interaction with the ordered QNU cations. Only at the highest temperature, some *d4r* cages in GeO_2_‐AST_QNU have a different orientation. Additionally, a few of the *d4r* cages in the 573 K average structures show no pyritohedral distortion at all, which probably indicates that a change in orientation occurs during the AIMD run of 7.5 ps. Although the published crystal structure of GeO_2_‐AST contains cube‐like *d4r* cages, it should be noted that the observed preference for distorted *d4r* cages agrees with the report of similarly distorted cages in the structurally related germanate ASU‐7.^[93]^ This raises the question to what extent such deformations are a general feature of *d4r*‐containing GeO_2_ zeotypes (or Ge‐rich zeolites). The coexistence of different orientations, together with thermal motion, would render it difficult to pin this down with diffraction methods.

##### 3.2.2.3 AlPO_4_‐AST

**Figure 7:**
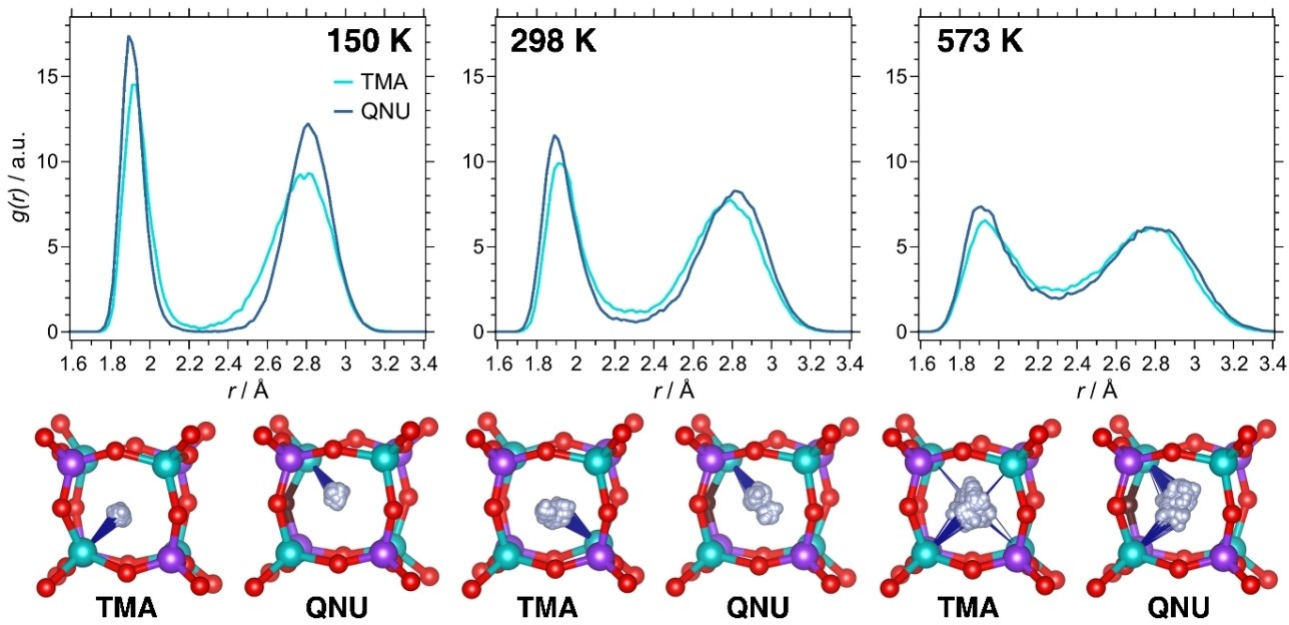
**Top**: F−Al RDFs in AlPO_4_‐AST_TMA and AlPO_4_‐AST_QNU. **Bottom**: Trajectories of individual fluoride anions. The positions of Al, P, and O atoms are taken from the respective average structures. Blue lines are used to indicate Al−F contacts below 2.0 Å.

The F−Al RDFs of AlPO_4_‐AST zeotypes, shown in Figure 7, exhibit very different features than those calculated for SiO_2_‐ and GeO_2_‐AST: Rather than having one broad maximum, there are two maxima, a sharp one centred at ∼1.9 Å, and a considerably broader one centred at ∼2.8 Å. A closer look at the trajectories of individual anions (Figure 7) and at the average structures (Figures S10a and S11a) confirms an off‐centre displacement of the fluoride anions towards one of the Al atoms at the corners of the *d4r* cage, leading to the formation of Al−F bonds. A re‐optimisation of AIMD average structures obtained at 150 K, and comparison to the initial structures with fluoride at the cage centre delivers energy differences of −2.2/−3.8 kJ mol^−1^ per AlPO_4_ formula unit (−11/−19 kJ mol^−1^ per fluoride anion) for AlPO_4_‐AST_TMA/AlPO_4_‐AST_QNU, corroborating that the formation of Al−F bonds is energetically favoured over a centre‐of‐cage position of fluoride. The optimised Al−F bond lengths of 1.90 Å agree with values observed in aluminophosphates where fluoride anions bridge between two Al atoms.[^53^, ^55^, ^57^]

**Figure 8:**
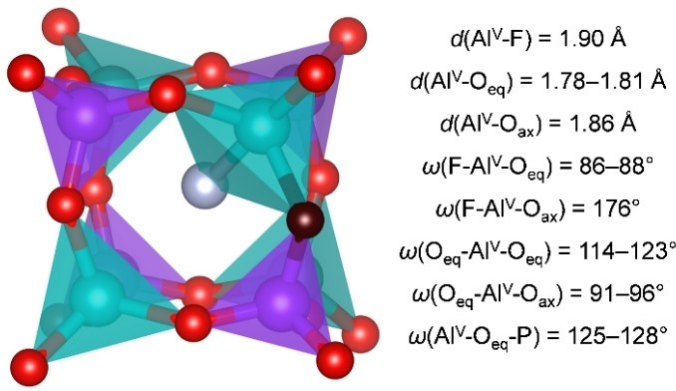
Representative *d4r* cage in AlPO_4_‐AST including an Al−F bond. Al^V^=Al atom in trigonal‐bipyramidal coordination, O_eq_=oxygen atoms forming the equatorial plane of the AlO_4_F trigonal bypramid, O_ax_=axial oxygen atom of the AlO_4_F trigonal bipyramid.

Due to the displacement of fluoride towards one Al corner, the coordination number of that Al atom increases to five, and an analysis of the distances and angles around a representative Al^V^ atom shows only relatively minor deviations from an ideal trigonal‐bipyramidal coordination environment (Figure 8). As is visible in the RDFs, the F−Al RDF in AlPO_4_‐AST_QNU falls to zero between the maxima at 150 K, indicating that all fluoride anions remain bonded to the same Al atom for the duration of the AIMD simulation. A certain, albeit very limited, mobility is observed in AlPO_4_‐AST_TMA at 150 K, and in both systems at 298 K (evidenced by non‐zero values between the two maxima, and by intermediate positions of some fluoride anions in the average structures). At 573 K, the mobility is enhanced considerably, but there are still two distinct maxima in the F−Al RDFs. This indicates that the fluoride anions remain bonded to Al atoms for the largest part of the simulation time, but move between different bonding partners on the picosecond timescale. This contrasts with the behaviour observed for SiO_2_‐AST and GeO_2_‐AST, where only oscillations around the cage centre occur. It is worth noting that the particular situation in AlPO_4_‐AST also explains the evolution of the F‐RMSDs with temperature: When most of the fluoride anions are bonded to a single Al atom during the whole AIMD simulation, their RMSD will be similar to that of the framework oxygen atoms due to the similar bond strength, as is indeed observed for 150 K. At higher temperature, the jumps between different Al atoms lead to a drastic increase in the RMSD values.

**Figure 9:**
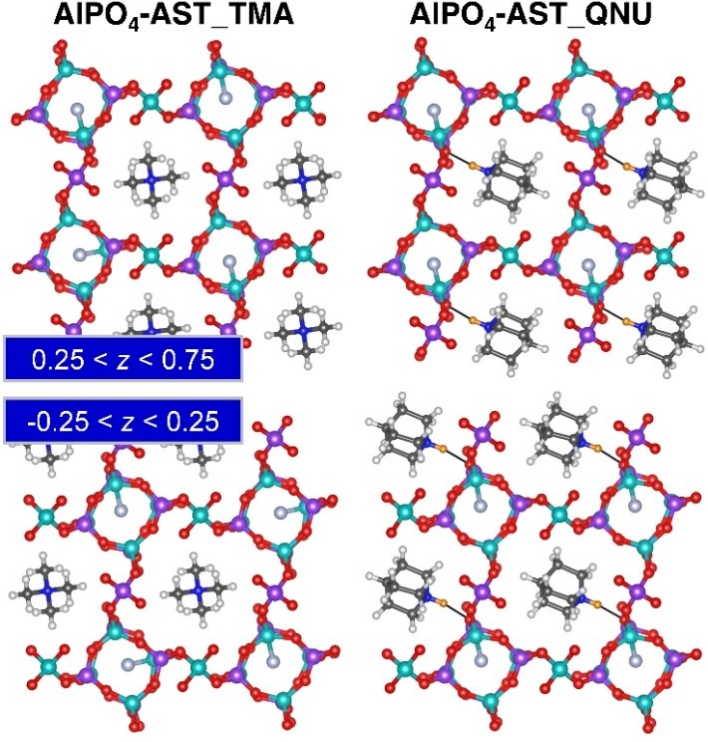
Representative average structures of AlPO_4_‐AST_TMA and AlPO_4_‐AST_QNU at 150 K. For clarity, different sections perpendicular to *c* are visualised separately.

While the formation of Al−F bonds occurs in both AlPO_4_‐AST_TMA and AlPO_4_‐AST_QNU, the nature of the OSDA has a pronounced effect on the direction of fluoride displacements: In the TMA‐containing system, the displacement pattern is random, without detectable preference for any of the four available Al atoms (Figure 9). The situation is different in AlPO_4_‐AST_QNU, where fluoride anions are preferentially bonded to the Al atom neighbouring the oxygen atom that acts as hydrogen bond acceptor. This preference is almost perfectly realised at 150 and 298 K (Figure S11a). It can be explained as a consequence of the local perturbations that are related to the N−H⋅⋅⋅O hydrogen bond: Because the oxygen atom participates in the hydrogen bond, electron density is withdrawn from the Al atom, leading to an increased positive polarisation which favours the formation of an Al−F bond. Additionally, the oxygen atom is somewhat displaced into the *ast* cage, rendering the Al atom more amenable to an expansion of its coordination environment to trigonal‐bipyramidal. The preferred direction of fluoride displacement resembles the ordering of the pyritohedron‐like *d4r* cages observed in GeO_2_‐AST_QNU: In both cases, the reduced symmetry of the OSDA, together with its ability to form hydrogen bonds, causes an energetic preference for a particular orientation of the fluoride‐containing *d4r* cages.

It is worth pointing out that neither previous crystallographic studies nor the DFT optimisations of the present work pointed to the presence of Al−F bonds. The inability of diffraction methods to observe this off‐centre displacement can be attributed to a) the lack of ordering of the displacements in real crystals, where the OSDA molecules are likely to exhibit orientational disorder, and b) the high mobility of the fluoride anions. It is worth noting that NMR studies of AlPO_4_‐16 synthesised in the presence of fluoride anions reported two signals in the ^19^F MAS NMR spectra, one of which was attributed to “terminal” fluoride anions.[^50^, ^84^] Tentatively, this signal could be due to fluoride anions that remain bonded to a single Al atom for relatively extended periods (on the timescale of NMR experiments). The fact that the DFT optimisations reported in 3.1 did not deliver Al−F bonds indicates that the structures with fluoride at the cage centre correspond to local minima on the potential energy surface, which the DFT optimisation algorithm cannot leave.

##### 3.2.2.4 GaPO_4_‐AST

**Figure 10:**
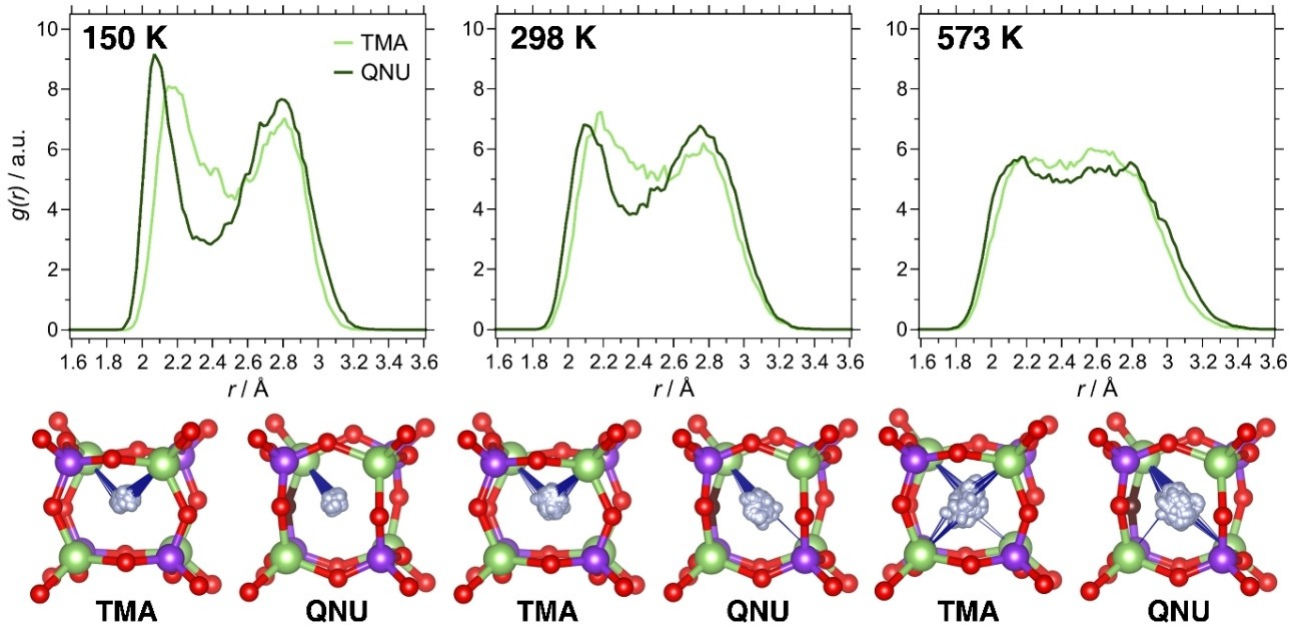
**Top**: F−Ga RDFs in GaPO_4_‐AST_TMA and GaPO_4_‐AST_QNU. **Bottom**: Trajectories of individual fluoride anions. The positions of Ga, P, and O atoms are taken from the respective average structures. Blue lines are used to indicate Ga−F contacts below 2.0 Å.

**Figure 11:**
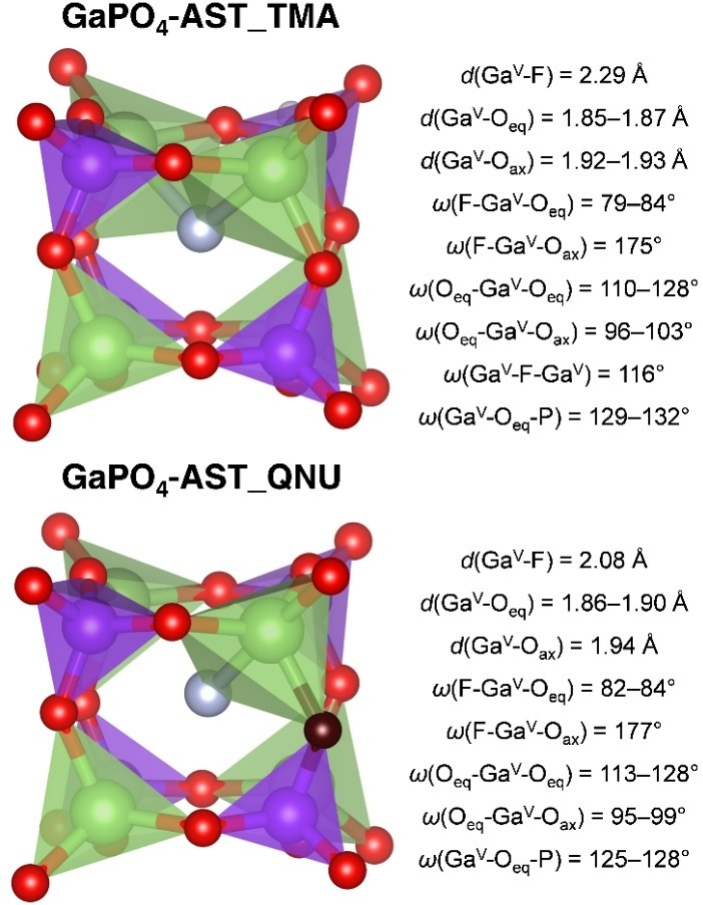
**Top**: Representative *d4r* cage in GaPO_4_‐AST_TMA with fluoride in a “bridging” position between two Ga atoms. **Bottom**: Representative *d4r* cage in GaPO_4_‐AST_QNU containing a single Ga−F bond.

The F−Ga RDFs obtained for GaPO_4_‐AST show two maxima, indicating an off‐centre displacement of the fluoride anions that is comparable to that observed in AlPO_4_‐AST (Figure 10). However, the maxima are less well separated, and significant *g*(*r*) values are observed between them even at 150 K, especially for GaPO_4_‐AST_TMA. A visualisation of the average structures (Figures S12a and S13a) shows that *d4r* cages in which fluoride is bonded to a single Ga atom are more typical in GaPO_4_‐AST_QNU, whereas configurations where fluoride is displaced towards one face with similar distances to two Ga atoms occur more frequently in GaPO_4_‐AST_TMA. This distinct difference, which is qualitatively visible in the visualisation of individual fluoride trajectories (lower part of Figure 10), also manifests as a shift of the first maximum in the F−Ga RDF towards lower distances in GaPO_4_‐AST_QNU. To corroborate this further, average structures obtained at 150 K were re‐optimised. For GaPO_4_‐AST_TMA, the energy difference with respect to the $I\bar{4}$ structure amounts to −2.7 kJ mol^−1^ per GaPO_4_ formula unit (−13.5 kJ mol^−1^ per fluoride anion). An inspection of the optimised structure shows many *d4r* cages with two Ga−F contacts of similar length (typically ∼2.3 Å), indicating that such “bridging” modes of fluoride correspond to local minima (Figure 11, top). An analogous calculation for GaPO_4_‐AST_QNU delivers an energy difference of −2.8 kJ mol^−1^ per GaPO_4_ formula unit (−14 kJ mol^−1^ per fluoride anion). In this case, configurations having a single Ga−F bond with a length of ∼2.08 Å dominate (Figure 11, bottom). This dependence of the equilibrium position of fluoride on the OSDA, together with the coexistence of different binding modes within the same structure, point to a very shallow potential energy surface with different local minima. Again, the DFT‐optimised Ga−F distances are close to those observed in experimental structures of systems containing Ga−F−Ga bridges.[^10^, ^54^]

In addition to the off‐centre displacement of the fluoride anions, the *d4r* cages in the GaPO_4_‐AST zeotypes also show a pyritohedron‐like distortion, as observed above for GeO_2_‐AST (bottom part of Figure 10). In the TMA‐containing system, neither fluoride displacements nor the distortions of the *d4r* cages are ordered in any way (Figure S12a). In GaPO_4_‐AST_QNU, the same displacement pattern of the fluoride anions as in the corresponding AlPO_4_‐AST system is observed, which, while being most pronounced at 150 K, persists up to 573 K (Figure S13a). In contrast, the distortions of the *d4r* cages have a random orientation, at variance with the findings for GeO_2_‐AST_QNU. Apparently, framework‐OSDA interactions have a larger influence on the preferential formation of Ga−F bonds than on the deformation of the *d4r* units in GaPO_4_‐AST, and the two phenomena are not strictly coupled to each other.

#### 3.2.3 OSDA Dynamics and Hydrogen Bonds

The final part of the analysis deals with the dynamic behaviour of the OSDA molecules, with special emphasis on the presence of hydrogen bonds and their evolution with temperature. Due to significant disorder of the OSDAs at 298 K and 573 K, the AIMD average structures deliver only a limited amount of information in this regard. The analysis presented here relies on the F−N and H−O RDFs, because these quantities provide a convenient means to assess the most relevant features of the OSDA dynamics. For the F−N RDFs, only fluoride anions and nitrogen atoms lying approximately in one plane perpendicular to the *c* axis were considered, leading to four, rather than six, F−N contacts in the distance range up to ∼10 Å (Figure 1).

**Figure 12:**
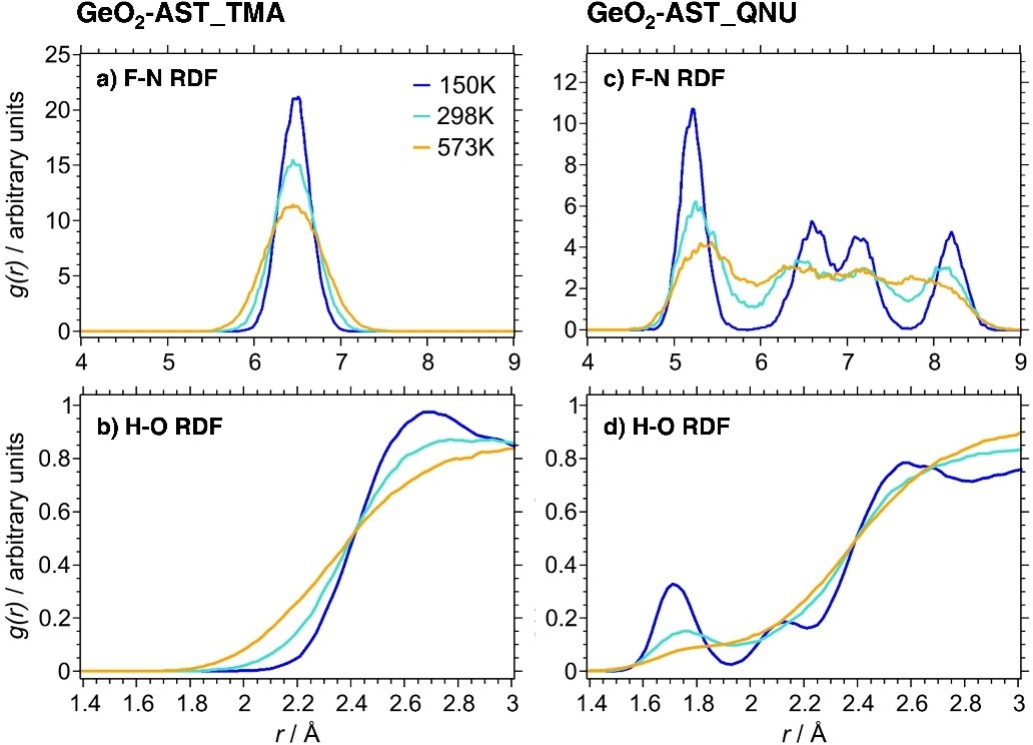
F−N and H−O RDFs of GeO_2_‐AST_TMA (left) and GeO_2_‐AST_QNU (right)

With the exception of the F−N RDF of AlPO_4_‐AST_TMA, which will be addressed separately below, the F−N and H−O RDFs of all other TMA‐containing systems show very similar features (see Supporting Information). They are visualised for the representative example of GeO_2_‐AST_TMA in Figure 12. The symmetric maximum in the F−N RDF, which broadens with temperature, can be attributed to oscillations of the TMA cations (and, to a lesser extent, the fluoride anions) about their equilibrium positions. The H−O RDF shows a broad shoulder that starts to rise at distances of about 2.1 to 2.2 Å at 150 K, and moves progressively towards lower distances with increasing temperature. In their combined DFT and NMR study of SiO_2_‐AST_TMA, Dib et al. concluded that weak C−H⋅⋅⋅O “hydrogen bonds” are formed between some TMA hydrogen atoms and framework oxygen atoms.^[69]^ If such bonds were present, it would be reasonable to expect an elongation of the H⋅⋅⋅O distance with increasing temperature, as increased thermal motion should weaken and – at sufficiently high temperature – break the bonds. The observation of shorter, rather than longer H⋅⋅⋅O distances upon increasing temperature provides no evidence for the presence of hydrogen bonds, indicating instead that non‐directional interactions dominate.

Since the features of F−N and H−O RDFs of the QNU‐containing systems having different framework composition are also qualitatively similar, the discussion will largely focus on the results obtained for GeO_2_‐AST_QNU (Figure 12). The F−N RDF obtained for 150 K shows four distinct maxima, which correspond to the distances from a given fluoride anion to the nitrogen atoms belonging to QNU cations in the four surrounding *ast* cages (Figure 1). With increasing temperature, the four maxima become increasingly blurred, leading to a single broad maximum with a poorly developed substructure at 573 K. The broadening of the F−N RDF is considerably more pronounced than that of the F−Ge RDF (Figure 5), which can be attributed to the increased thermal motion of the OSDA cations. This is corroborated when looking at the H−O RDFs: At 150 K, there is a distinct first maximum centred at ∼1.7 Å, together with a second, smaller maximum centred at ∼2.1 Å. The respective positions of these maxima agree with the lengths of the N−H⋅⋅⋅O/C−H⋅⋅⋅O hydrogen bonds from the terminal H atoms of the QNU molecule to framework O atoms in the DFT‐optimised structure (Figure S2). A third, broad maximum at ∼2.6 Å corresponds to other H⋅⋅⋅O contacts. With increasing temperature, the first maximum decreases significantly, and the second maximum disappears altogether (the first maximum also disappears in other QNU‐containing systems at 573 K). This indicates that the hydrogen bonds are weakened, and eventually broken, due to more vigorous movements of the QNU cations. Once the hydrogen bonds are broken, the OSDA molecules are prone to reorient within the *ast* cages. Indeed, the visualisation of representative last AIMD frames obtained for SiO_2_‐AST_QNU and GeO_2_‐AST_QNU (for *T*=573 K) shows that several QNU cations have changed their orientation with respect to their perfect ordering in the starting structures (Figure S14). Given the impact of framework‐OSDA interactions on the ordering of pyritohedral distortions in GeO_2_‐ and GaPO_4_‐AST_QNU, and on the ordered displacements of fluoride anions in AlPO_4_‐ and GaPO_4_‐AST_QNU, it is straightforward to link the reorientation of some QNU cations to a reduced degree of ordering at 298 K and, especially, 573 K. The pronounced orientational disorder of the OSDA in the experimental crystal structures of SiO_2_‐AST_QNU and AlPO_4_‐AST_QNU (AlPO_4_‐16), obtained at room temperature, agrees with these AIMD results.[^11^, ^50^]

Finally, it is worth taking a separate look at the F−N RDFs of AlPO_4_‐AST_TMA and AlPO_4_‐AST_QNU. In the former system, the F−N RDF shows a distinct two‐peak maximum at 150 K that develops into an unusually broad maximum at higher temperatures (Figure S10d). This observation is clearly related to off‐centre displacements of the fluoride anions towards Al atoms, which lead to shorter and longer distances to the central nitrogen atoms of the surrounding TMA molecules. In the case of AlPO_4_‐AST_QNU, the F−N RDF obtained for 150 K shows a first main maximum centred at ∼4.9 Å and a secondary maximum centred at ∼5.3 Å. This secondary maximum stems from those local environments where fluoride anions are displaced towards Al atoms that do not neighbour the hydrogen bond acceptor O atom. Although these displacements account for only a minor fraction of all fluoride anions (2 out of 24 fluoride anions in the 150 K average structures, Figure S11a), their contribution is clearly visible in the F−N RDF. Even at the lowest temperature considered, the ordered displacement of the fluoride anions imposed by the ordering of QNU cations is not strictly realised.

## 4 Conclusions

The AIMD simulations have revealed a distinctly different behaviour of the fluoride‐containing *d4r* units depending on the framework composition: In SiO_2_‐AST, the fluoride anions oscillate about the cage centre, and the *d4r* cages retain their cube‐like shape (apart from transient distortions). Although the fluoride anions possess a larger freedom of motion in GeO_2_‐AST due to the larger dimensions of the cage, their average positions remain close to the cage centre. However, the *d4r* cages show a pronounced pyritohedral distortion, which goes hand‐in‐hand with a concerted rotation of the GeO_4_ tetrahedra and a narrower distribution of the Ge−O−Ge angles. In AlPO_4_‐AST, the fluoride anions are displaced towards one of the Al corners of the cage, forming Al−F bonds having a length of ∼1.9 Å. Apart from the formation of an AlO_4_F trigonal bipyramid at one corner, the *d4r* cages are only slightly distorted. Finally, GaPO_4_‐AST combines both phenomena observed in GeO_2_‐AST and AlPO_4_‐AST, exhibiting both a pyritohedron‐like distortion and a displacement of fluoride anions towards one or two Ga corners. Although these distortions occur, in the first place, independently of the nature of the OSDA, pronounced differences are observed with regard to the ordering of the distorted configurations: If the structures contain highly symmetric TMA cations, for which non‐directional interactions with the framework dominate, the distortions occur in an essentially random fashion. If less symmetric QNU cations are incorporated, these form (at least) one hydrogen bond to the framework. An ordered arrangement of the QNU cations, assumed in the starting structures, then leads to an ordered pattern of hydrogen bonds which causes, in turn, an ordering of the pyritohedron‐like distortions (GeO_2_‐AST) or the displacements of fluoride anions towards one corner of the cage (AlPO_4_‐AST, GaPO_4_‐AST). In other words, ordering of the OSDAs triggers collective deformations of the framework. The degree of ordering decreases with temperature due to increased thermal motion.

It has to be conceded that the assumption of a fully ordered arrangement of the QNU cations is a significant approximation. It cannot be expected that such a strict ordering would occur in real AST‐type zeotypes, which are typically synthesised at temperatures of 450 to 480 K, where thermal motion of the OSDAs will be considerable.[^46^, ^50^, ^83^] Nevertheless, the present work provides important new insights into the local structure of these systems, and it highlights the theoretical possibility to induce collective framework deformations through an ordering of the extra‐framework species. It could be envisaged to judiciously choose other OSDAs that stabilise such distortions, which could be proposed on the basis of further computations. External fields might provide another possibility to induce an ordering of the OSDAs. While this work is not aimed at the prediction of any specific property of the investigated zeotypes, it can be anticipated that some material properties could be tuned through ordered distortions, which might be relevant for potential applications (*e. g*., in dielectrics).

It is worth emphasising that DFT optimisations starting from symmetric structures did not reveal the majority of local distortions, which became apparent only in the AIMD simulations. Except in the case of GeO_2_‐AST_QNU, the undistorted structures are local minima, and the optimisations will not leave these local minima. This highlights that it is advisable to either employ AIMD simulations, or – if these are not feasible – to generate a set of perturbed starting structures prior to the DFT optimisations in cases where local distortions are expected.

Finally, it is useful to revisit previous experimental findings in the context of the present work, and to discuss possible ways to verify the findings experimentally. With regard to GeO_2_ zeotypes, some previous studies have already shown a tendency of the *d4*r units to distort.[^44^, ^47^, ^49^, ^93^] This distortion is sometimes visible as a disorder of the edge oxygen atoms over different positions. On the other hand, the formation of short Al−F or Ga−F bonds has, so far, not been observed in AlPO_4_ or GaPO_4_ zeotypes where fluoride anions are encapsulated in the *d4r* cages. Clearly, the lack of long‐range ordering in real systems will hamper a detection of such bonding scenarios with (powder) diffraction methods, as the occupancy of the fourfold‐disordered F position would only amount to 0.25. The highly dynamic behaviour at room temperature might render it difficult to detect Al−F/Ga−F bonds with solid‐state NMR methods, but investigations at cryogenic temperatures could provide more insights. Furthermore, it could be interesting to assess whether the presence of these bonds should give rise to distinct signals in the vibrational spectra, which could possibly serve as “fingerprints”. In any event, the partly unexpected findings of the present study show that it should be worthwhile to revisit these zeotypes with state‐of‐the‐art experimental methods.

## Supporting Information

The Supporting Information to this article includes a PDF file providing additional figures (details of DFT‐optimised structures, AIMD average structures, radial distribution functions).

An EXCEL file including RMSDs and RDFs and ZIP archives containing sample CP2K input files, DFT‐optimised structures (in CIF format), AIMD trajectories (in PDB format), and AIMD average structures (in CIF format) have been deposited on Figshare: https://doi.org/10.6084/m9.figshare.12981557.v1.

## Conflict of interest

The authors declare no conflict of interest.

## Supporting information

As a service to our authors and readers, this journal provides supporting information supplied by the authors. Such materials are peer reviewed and may be re‐organized for online delivery, but are not copy‐edited or typeset. Technical support issues arising from supporting information (other than missing files) should be addressed to the authors.

Supplementary
