# Supplementary material for: Local Distortions in a Prototypical Zeolite Framework Containing Double Four‐Ring Cages: The Role of Framework Composition and Organic Guests
Source: Chemphyschem. 2020 Nov 27;22(1):40–54. doi: 10.1002/cphc.202000863 (PMC7839729; doi:10.1002/cphc.202000863)
Supplement: Supplementary file 1 — Supplementary [file CPHC-22-40-s001.pdf]

ChemPhysChem

Supporting Information

Local Distortions in a Prototypical Zeolite Framework Containing Double Four-Ring Cages: The Role of Framework Composition and Organic Guests**

Michael Fischer* and Linus Freymann

Content Page

SI1) Additional figures of DFT-optimised structures **S2**

SI2) Root mean square displacements **S7**

SI3) AIMD average structures and radial distribution functions **S8**

SI4) Overview of other supplementary files **S30**

SI1) Additional figures of DFT-optimised structures

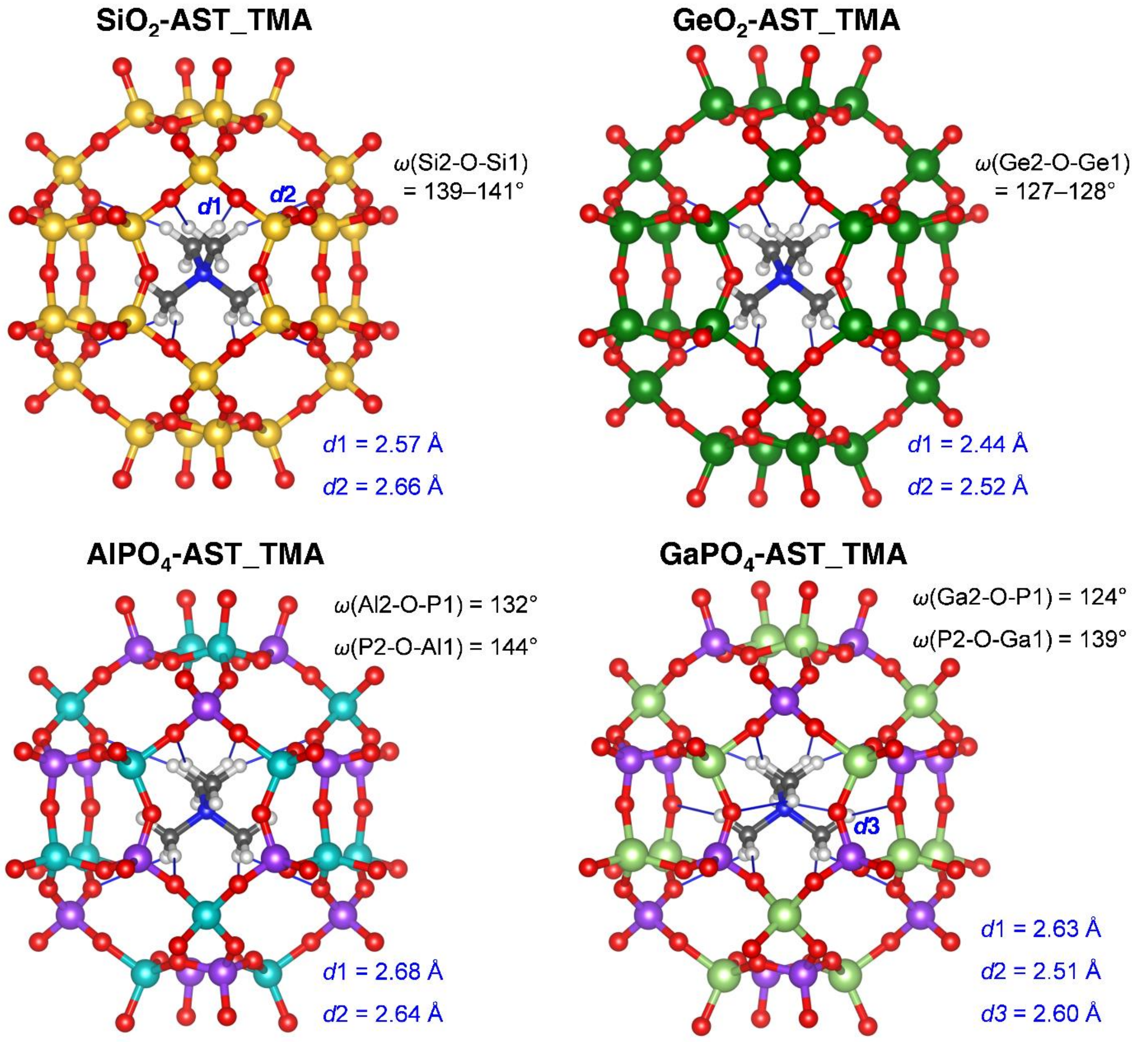

Figure S1: *ast* cages in DFT-optimised structures of AST_TMA zeotypes. Contacts between methyl hydrogen atoms and framework oxygen atoms are shown as thin blue lines if shorter than 2.70 Å.

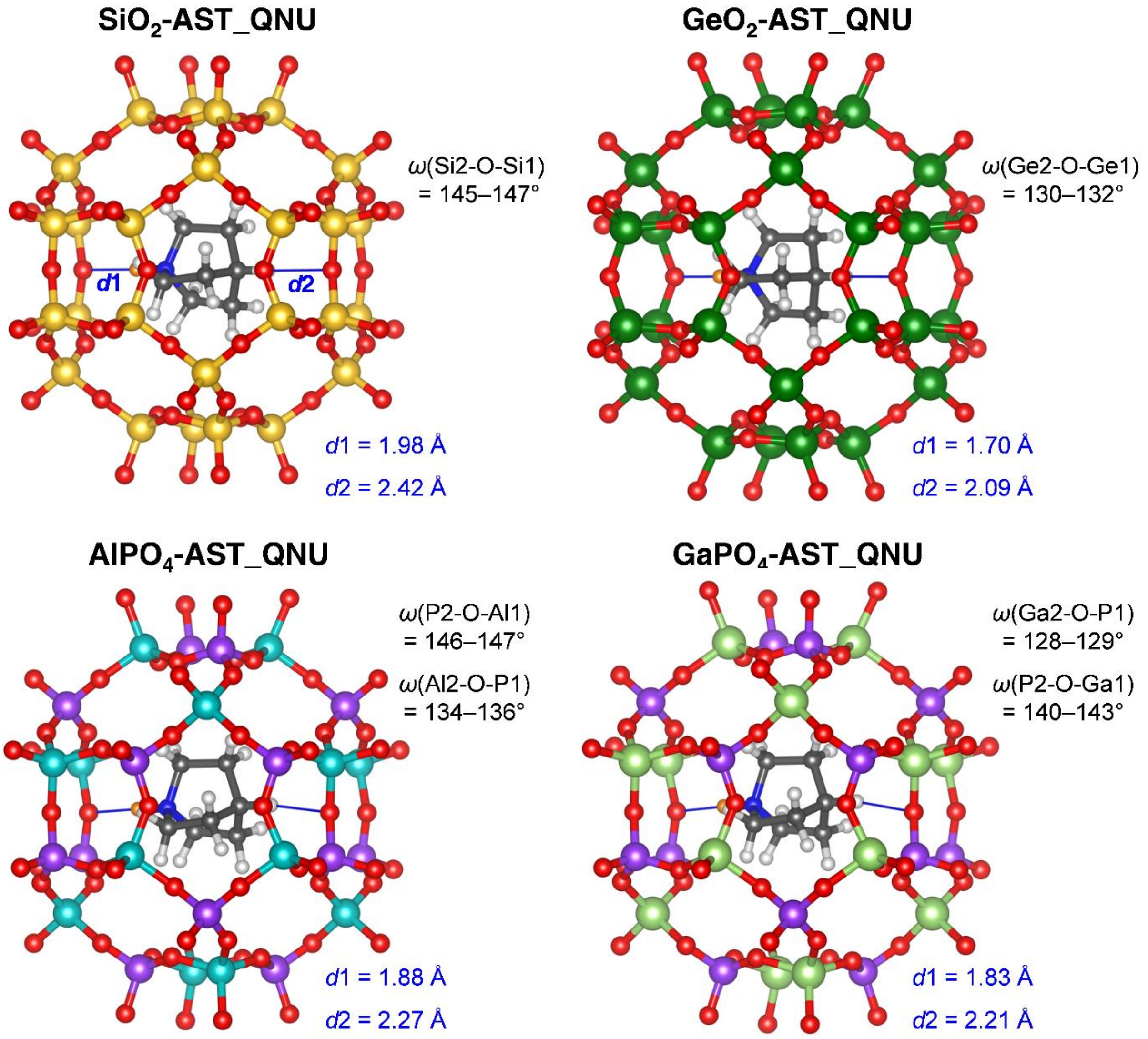

Figure S2: *ast* cages in DFT-optimised structures of AST_QNU zeotypes. Contacts between the terminal hydrogen atoms of QNU and closest framework oxygen atoms are indicated with thin blue lines.

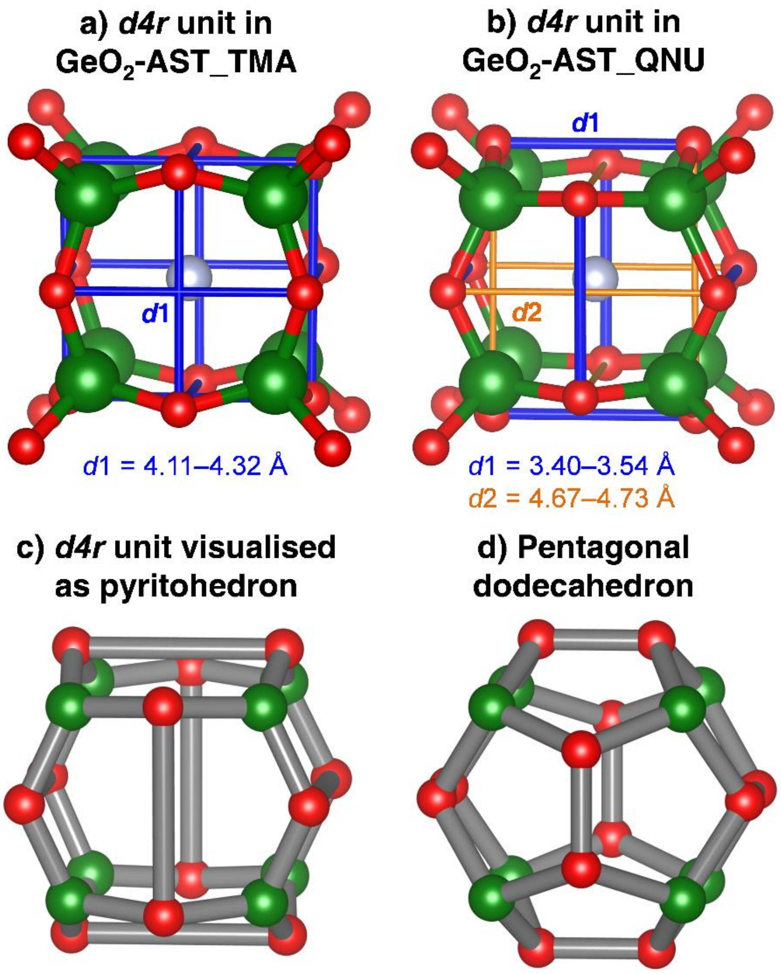

Figure S3: **a)** and **b)** *d4r* units in GeO_2-AST_TMA and GeO_2-AST_QNU, O-O distances across the faces are indicated by coloured lines; **c)** visualisation of the *d4r* unit of **b)** with Ge-O bonds and short O-O contacts shown in the same colour to emphasise the pyritohedron-like shape; **d)** perfect pentagonal dodecahedron (for comparison).

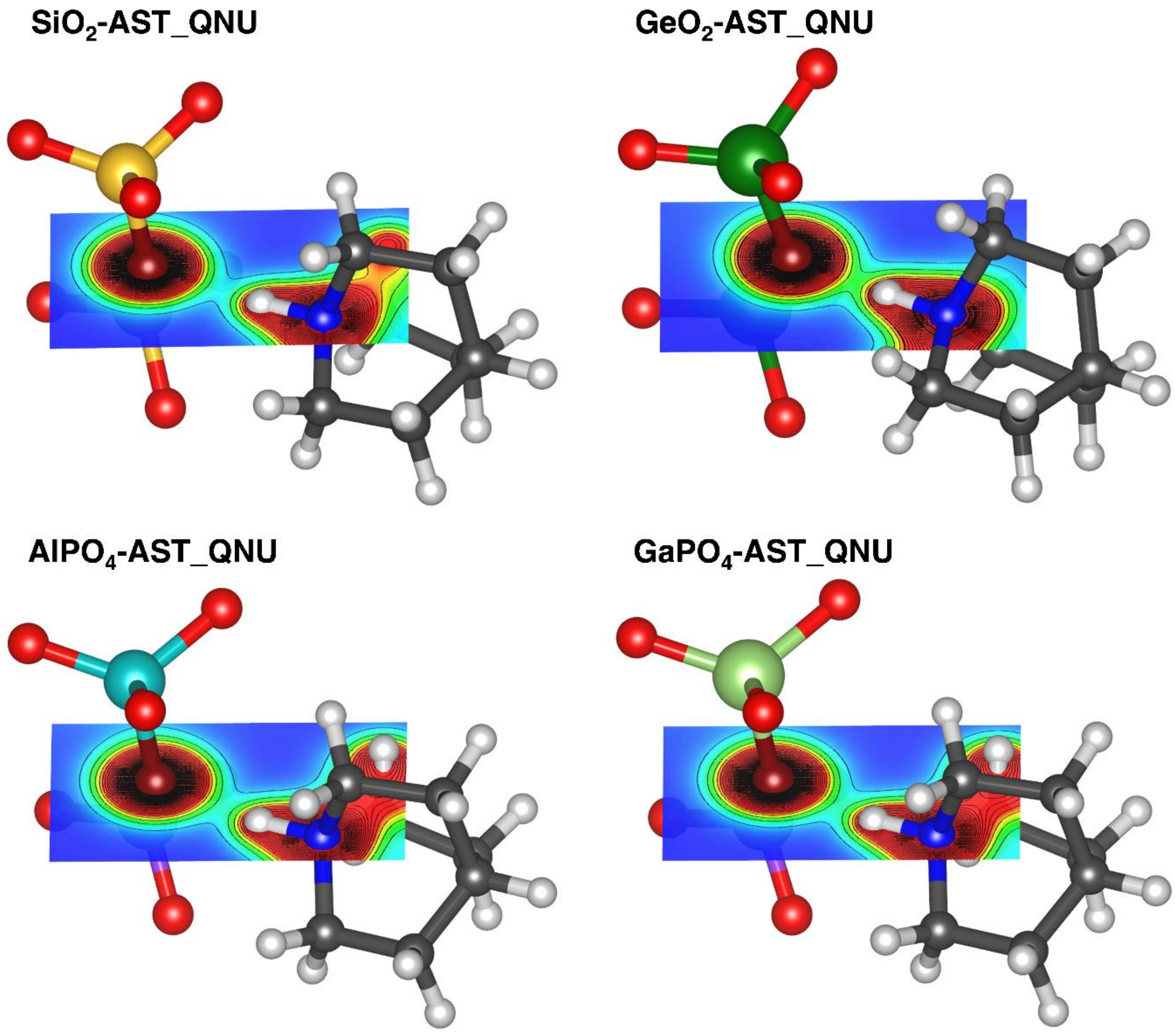

Figure S4: Valence electron density maps showing the vicinity of the N-H···O hydrogen bond in AST_QNU zeotypes. Isolines are drawn using a spacing of 0.02 *e* $bohr^{-3}$.

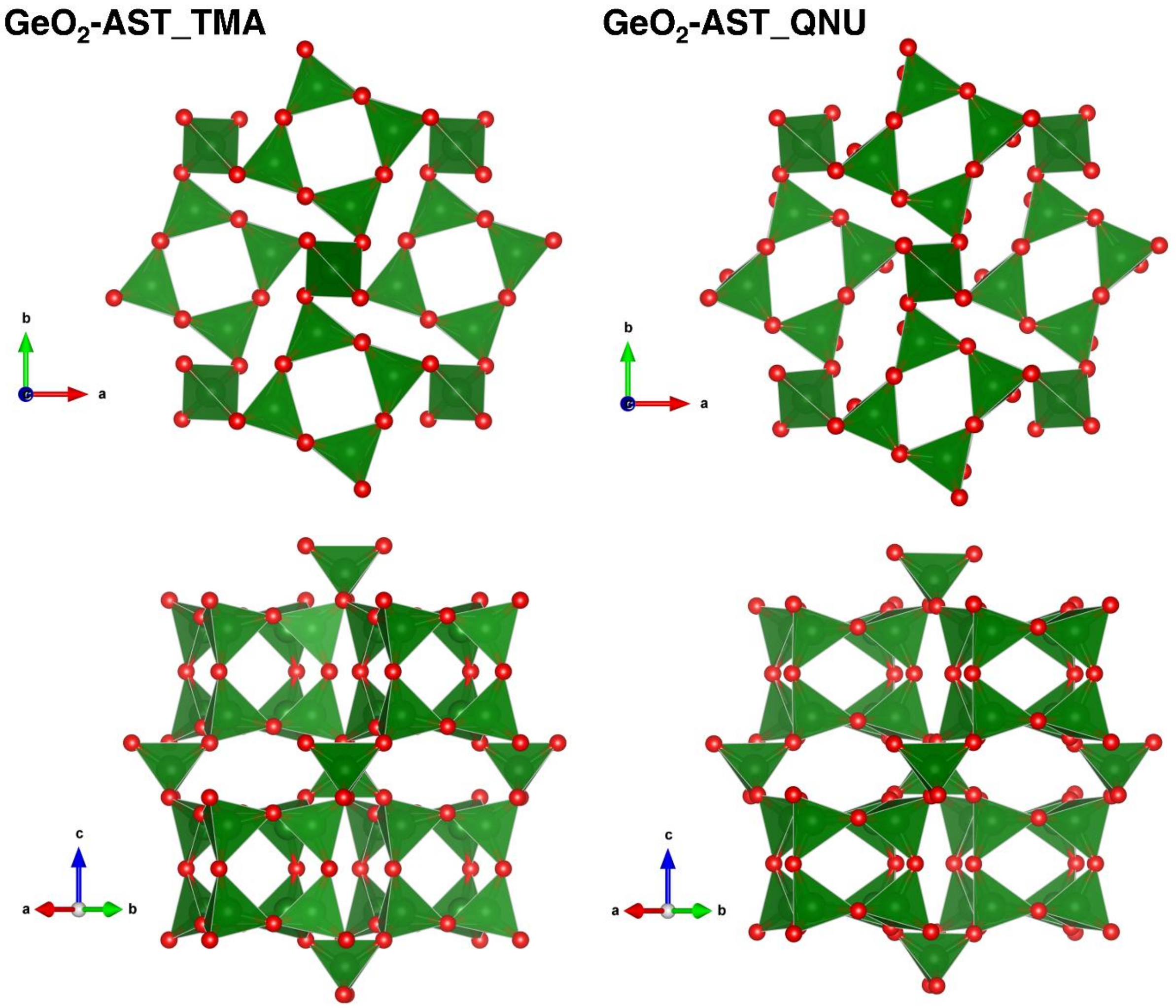

Figure S5: Framework structures of GeO_2-AST_TMA and GeO_2-AST_QNU. **Top:** view along [001], **bottom:** view along [110].

SI2) Root mean square displacements

Table S1: Root mean square displacements of T, O, and F atoms (all values in Å)

	**OSDA**	**TMA**	**TMA**	**TMA**	**QNU**	**QNU**	**QNU**
	T	**150 K**	**298 K**	**573 K**	**150 K**	**298 K**	**573 K**
**SiO_2-AST**	**RMSD(Si)**	0.09±0.01	0.12±0.01	0.17±0.01	0.09±0.01	0.15±0.02	0.17±0.02
	**RMSD(O)**	0.17±0.02	0.20±0.02	0.26±0.02	0.14±0.01	0.21±0.02	0.26±0.02
	**RMSD(F)**	0.18±0.03	0.27±0.04	0.37±0.05	0.19±0.03	0.29±0.04	0.39±0.05
**GeO_2-AST**	**RMSD(Ge)**	0.11±0.01	0.15±0.01	0.20±0.01	0.10±0.01	0.18±0.02	0.20±0.02
	**RMSD(O)**	0.15±0.01	0.20±0.01	0.32±0.03	0.14±0.01	0.23±0.02	0.30±0.02
	**RMSD(F)**	0.26±0.04	0.35±0.05	0.44±0.05	0.26±0.04	0.35±0.05	0.43±0.06
**$AlPO_4$-AST**	**RMSD(Al)**	0.10±0.01	0.16±0.02	0.22±0.02	0.10±0.01	0.17±0.02	0.23±0.03
	**RMSD(P)**	0.10±0.01	0.14±0.01	0.20±0.02	0.10±0.01	0.16±0.02	0.21±0.02
	**RMSD(O)**	0.17±0.02	0.23±0.02	0.30±0.02	0.17±0.01	0.23±0.02	0.30±0.02
	**RMSD(F)**	0.18±0.04	0.31±0.05	0.45±0.05	0.16±0.04	0.30±0.07	0.43±0.07
**$GaPO_4$-AST**	**RMSD(Ga)**	0.13±0.02	0.18±0.02	0.24±0.02	0.13±0.01	0.17±0.01	0.24±0.02
	**RMSD(P)**	0.12±0.02	0.17±0.02	0.22±0.02	0.11±0.01	0.16±0.02	0.22±0.02
	**RMSD(O)**	0.16±0.01	0.24±0.03	0.36±0.03	0.17±0.01	0.24±0.02	0.36±0.03
	**RMSD(F)**	0.23±0.04	0.31±0.04	0.39±0.05	0.21±0.03	0.29±0.04	0.40±0.05

SI3) AIMD average structures and radial distribution functions

The following pages show average structures obtained from the AIMD trajectories as well as F-Si, F-N, and H-O radial distribution functions. In the visualisation of average structures, the OSDA molecules are omitted for clarity. Bonds between fluoride anions and framework atoms were visualised on the basis of a distance-based search that employed the following criteria:

- Si-F bonds were searched using a cutoff distance of 2.4 Å *(such bonds do not occur)*
- Ge-F bonds were searched using a cutoff distance of 2.5 Å *(such bonds do not occur)*
- Al-F bonds were searched using a first cutoff distance of 2.2 Å and a second cutoff distance of 2.4 Å. Bonds ≤ 2.2 Å are shown as thick bicolour lines, bonds between 2.2 and 2.4 Å are shown as thin black lines.
- Ga-F bonds were searched using a first cutoff distance of 2.3 Å and a second cutoff distance of 2.5 Å. Bonds ≤ 2.3 Å are shown as thick bicolour lines, bonds between 2.3 and 2.5 Å are shown as thin black lines.

All RDF plots correspond to averages over three independent trajectories. In each plot, the bottom part shows the radial distribution function *g(r)*, whereas the top part shows the integrated (cumulative) *g(r)*. The F-N RDFs consider only the four nearest neighbours, which correspond to sets of F and N atoms lying approximately in the same plane perpendicular to the *c* axis (see **Figure 1** of main paper).

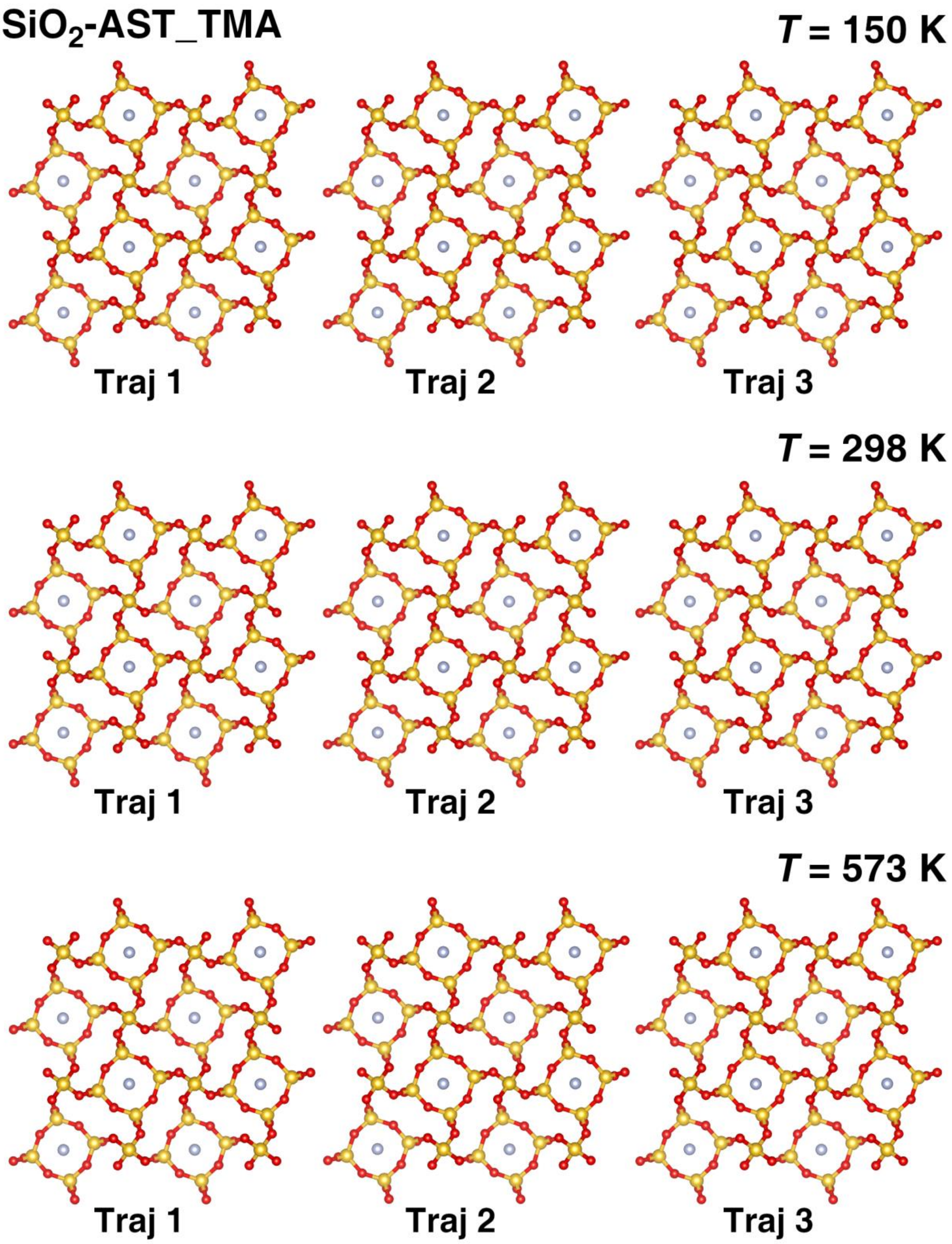

Figure S6a: Average structures of SiO_2-AST_TMA obtained from independent AIMD runs (framework + fluoride anions).

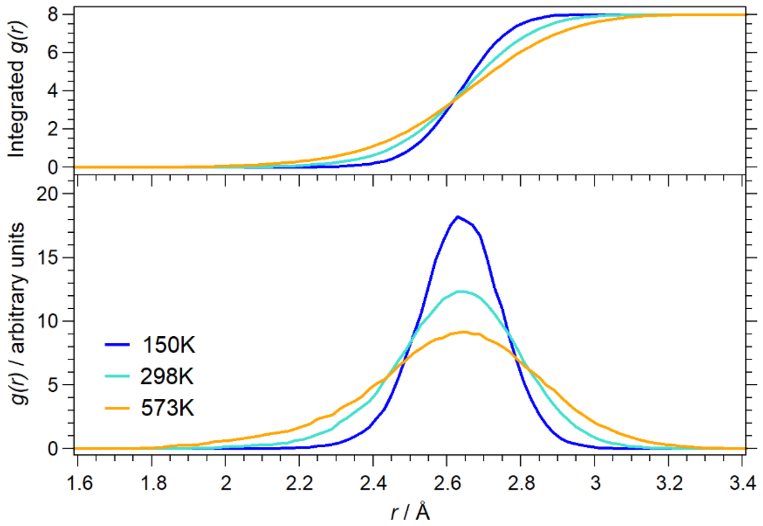

Figure S6b: F-Si RDFs of SiO_2-AST_TMA

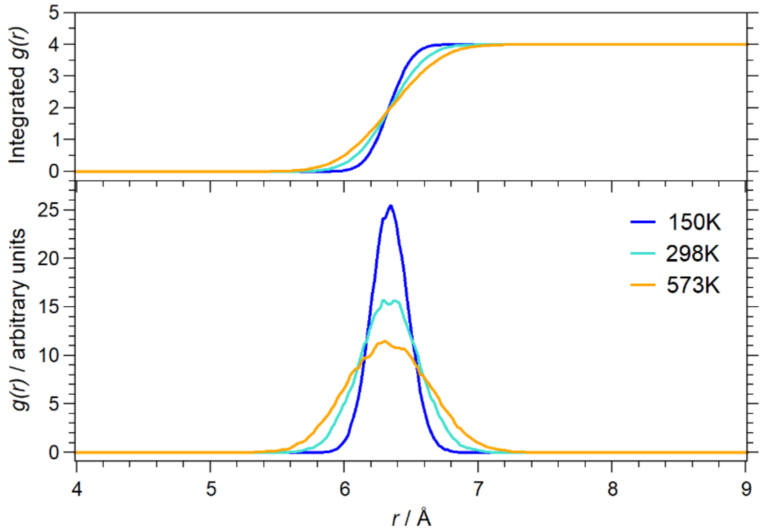

Figure S6c: F-N RDFs of SiO_2-AST_TMA

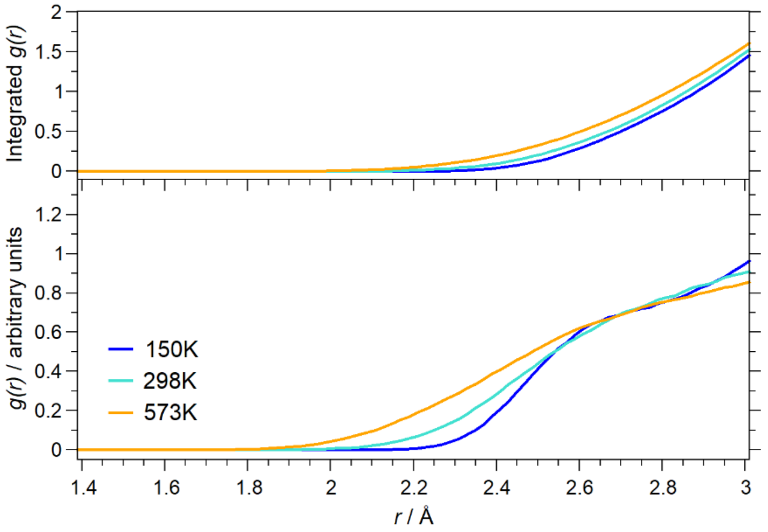

Figure S6d: H-O RDFs of SiO_2-AST_TMA

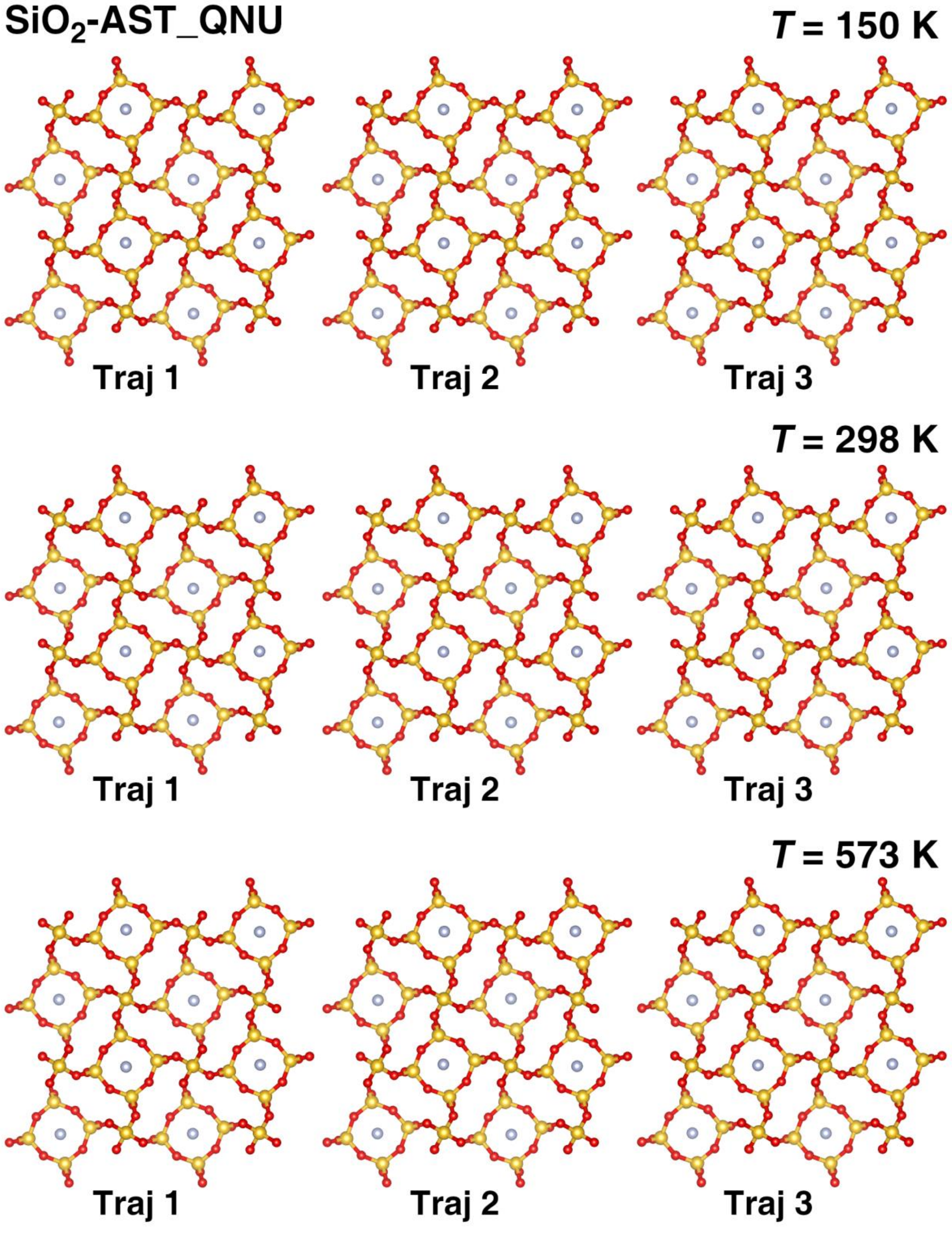

Figure S7a: Average structures of SiO_2-AST_QNU obtained from independent AIMD runs (framework + fluoride anions).

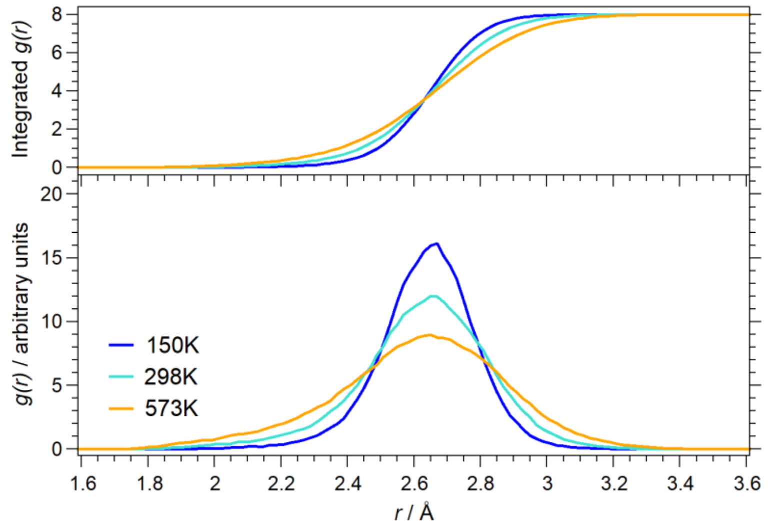

Figure S7b: F-Si RDFs of SiO_2-AST_QNU

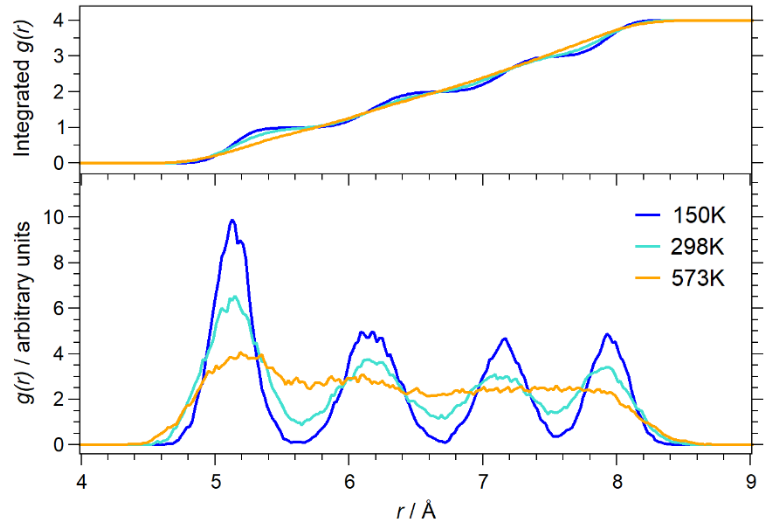

Figure S7c: F-N RDFs of SiO_2-AST_QNU

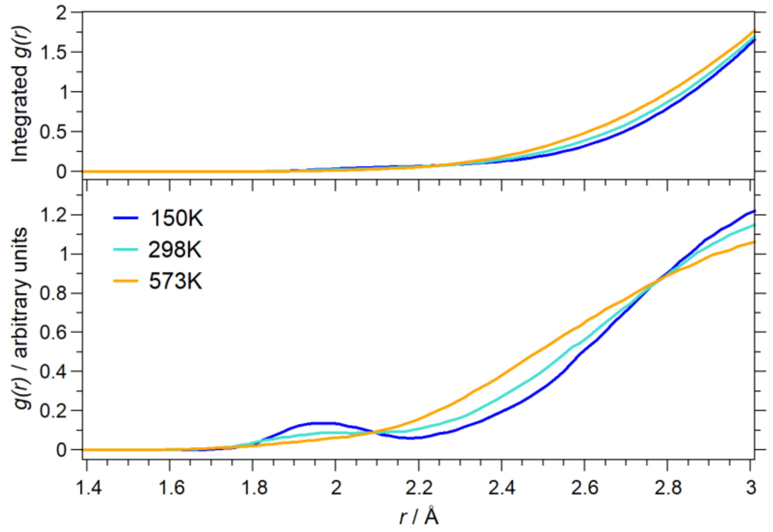

Figure S7d: H-O RDFs of SiO_2-AST_QNU

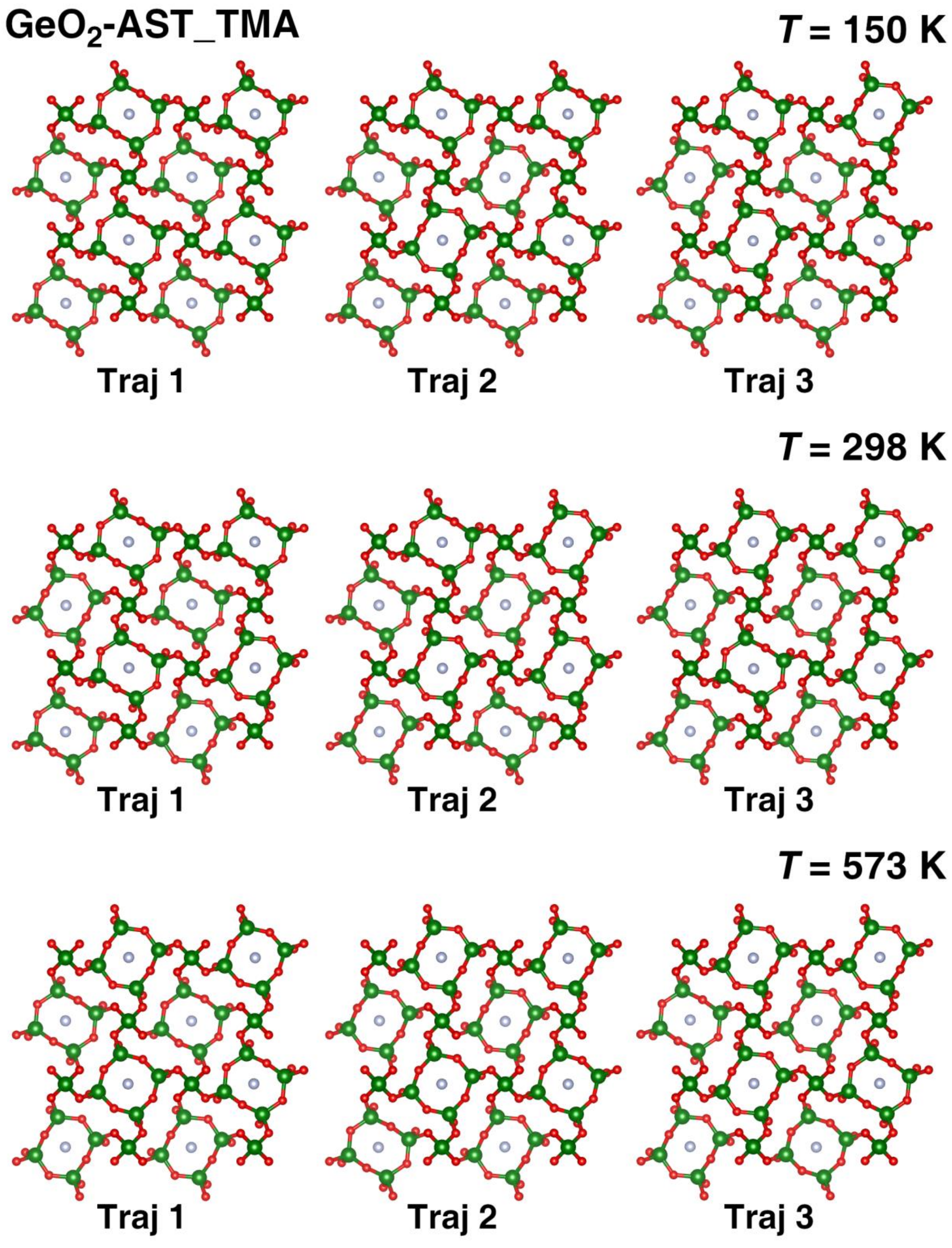

Figure S8a: Average structures of GeO_2-AST_TMA obtained from independent AIMD runs (framework + fluoride anions).

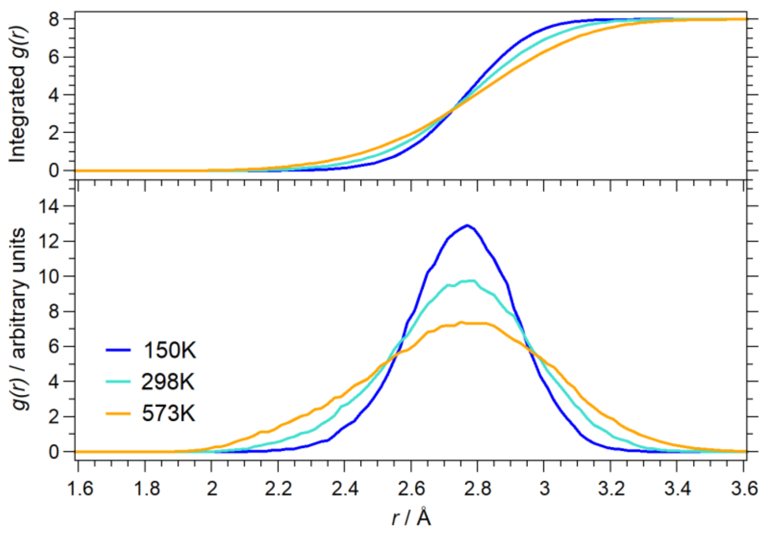

Figure S8b: F-Ge RDFs of GeO_2-AST_TMA

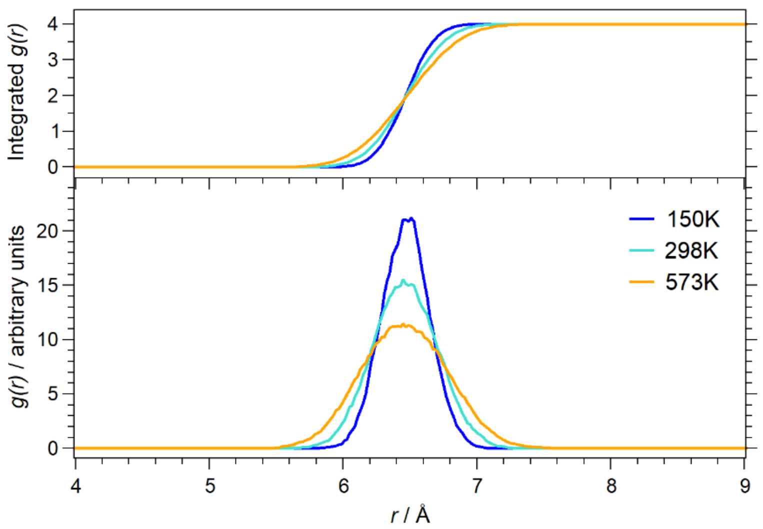

Figure S8c: F-N RDFs of GeO_2-AST_TMA

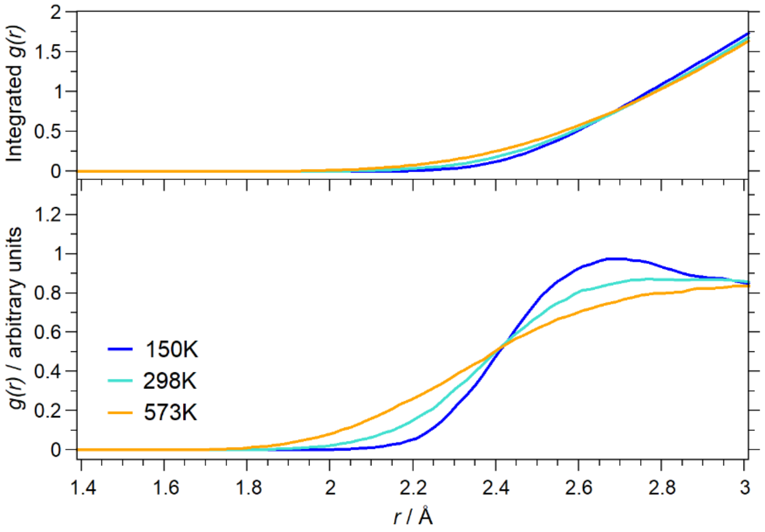

Figure S8d: H-O RDFs of GeO_2-AST_TMA

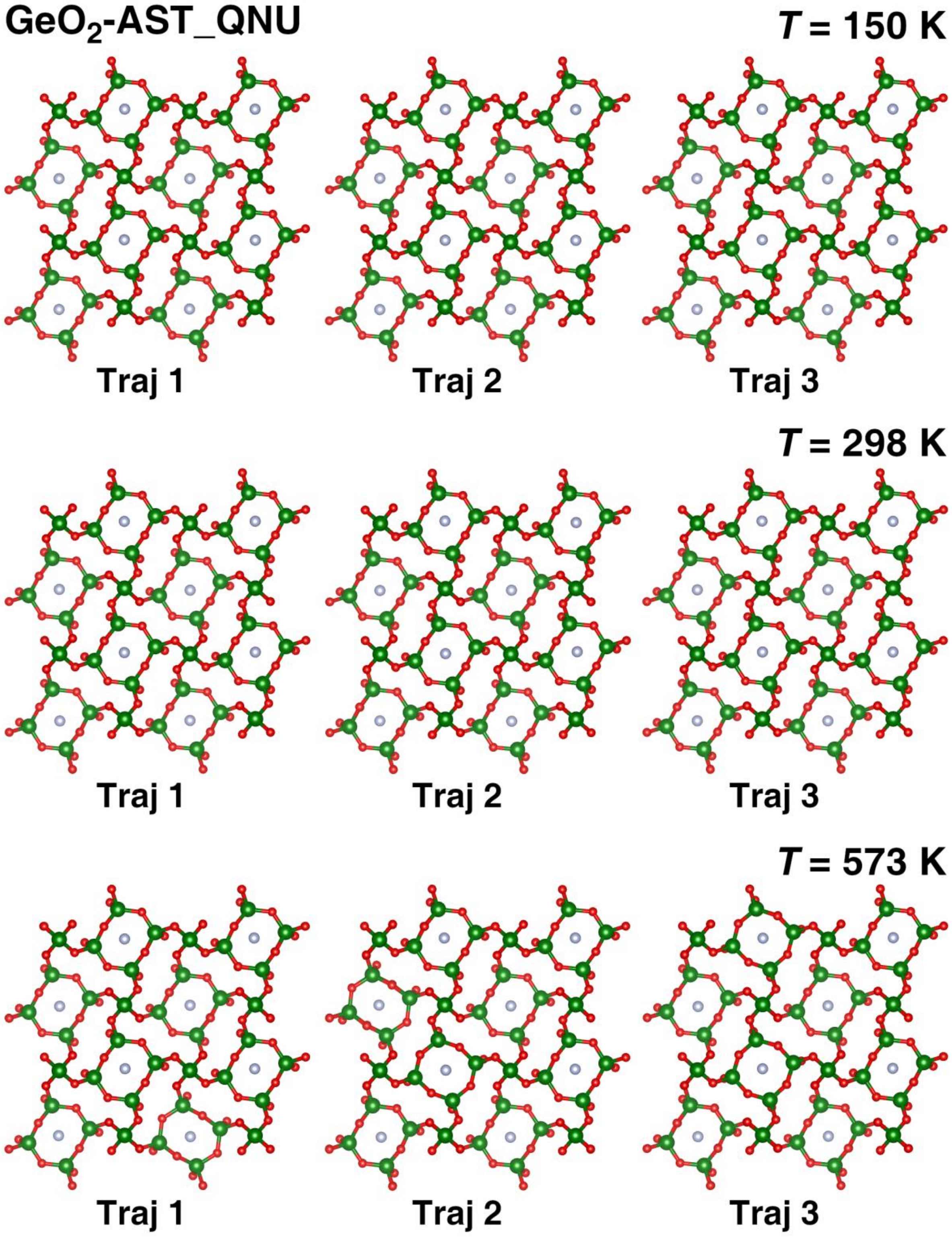

Figure S9a: Average structures of GeO_2-AST_QNU obtained from independent AIMD runs (framework + fluoride anions).

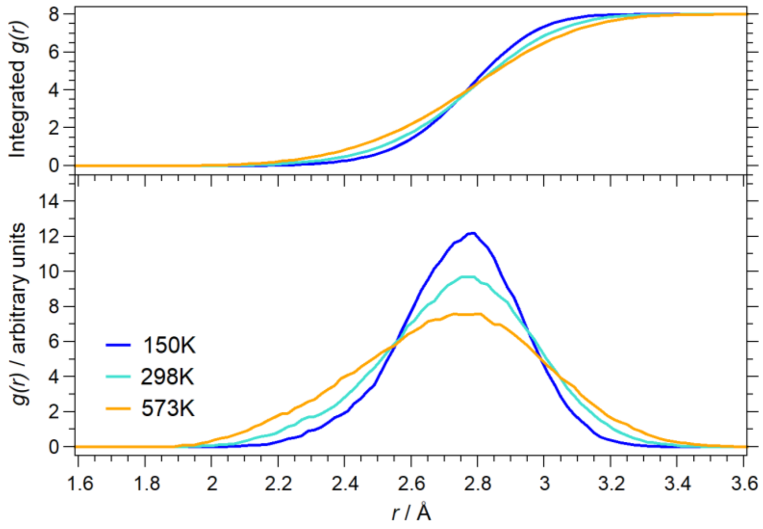

Figure S9b: F-Ge RDFs of GeO_2-AST_QNU

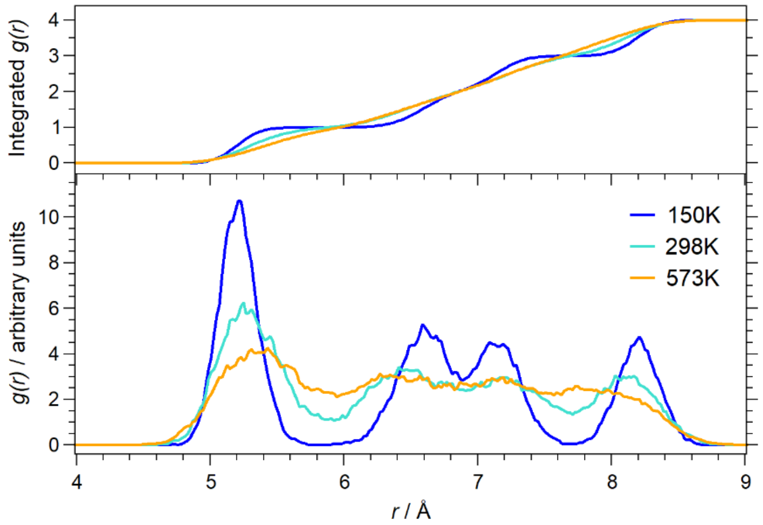

Figure S9c: F-N RDFs of GeO_2-AST_QNU

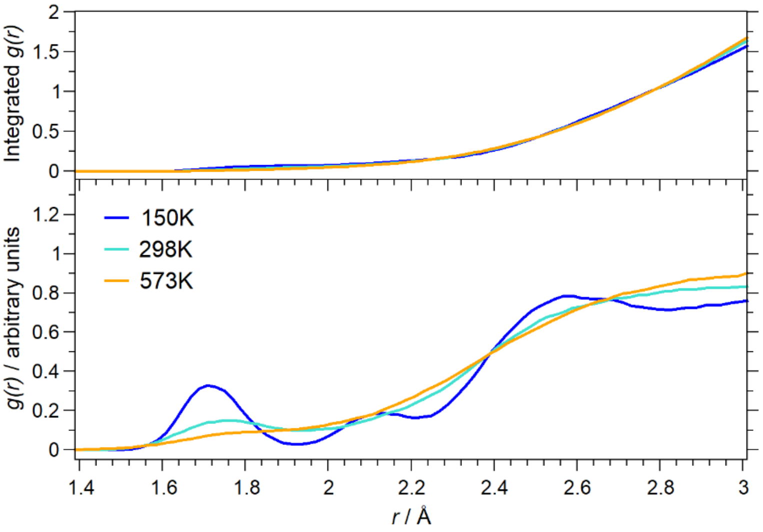

Figure S9d: H-O RDFs of GeO_2-AST_QNU

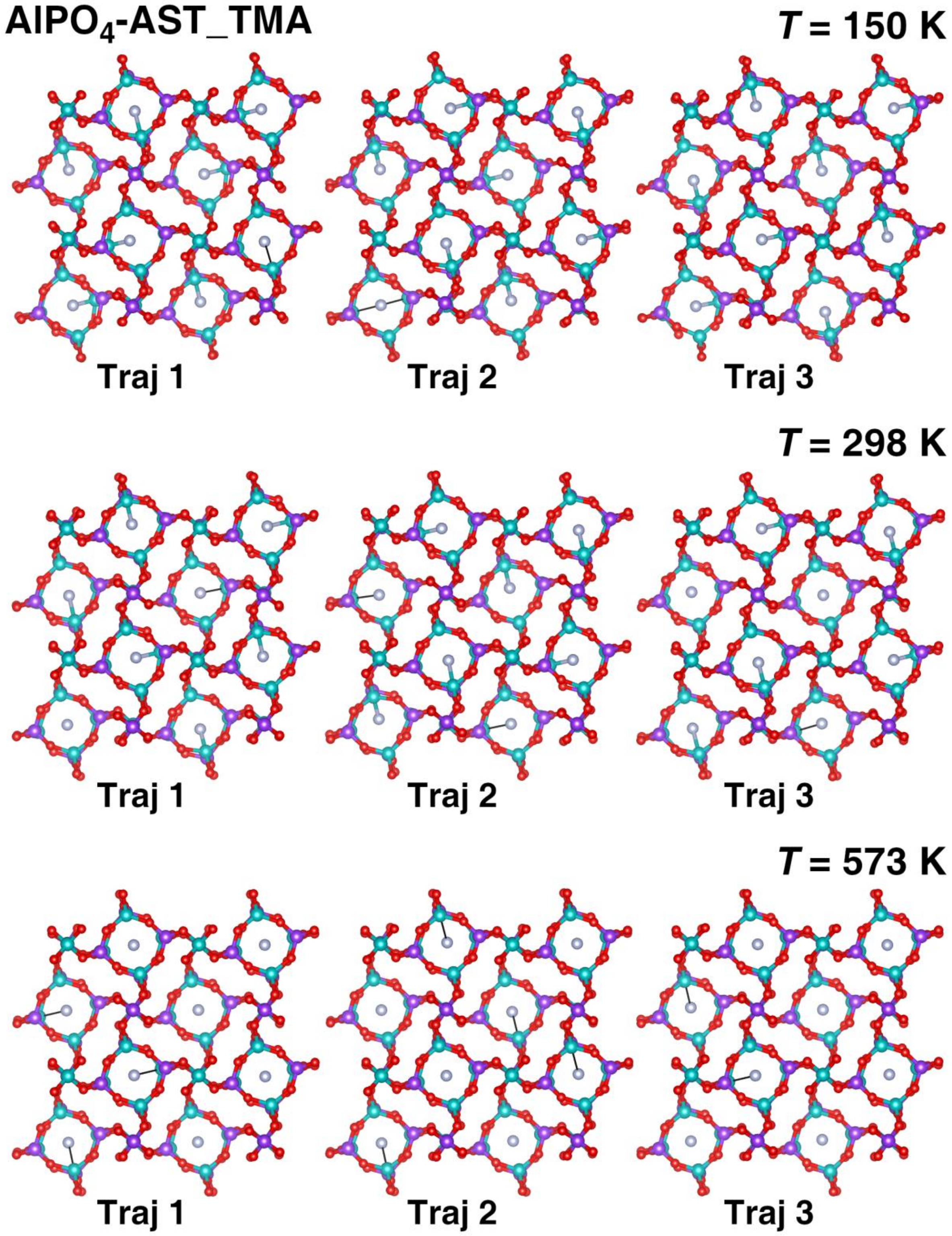

Figure S10a: Average structures of $AlPO_4$-AST_TMA obtained from independent AIMD runs (framework + fluoride anions).

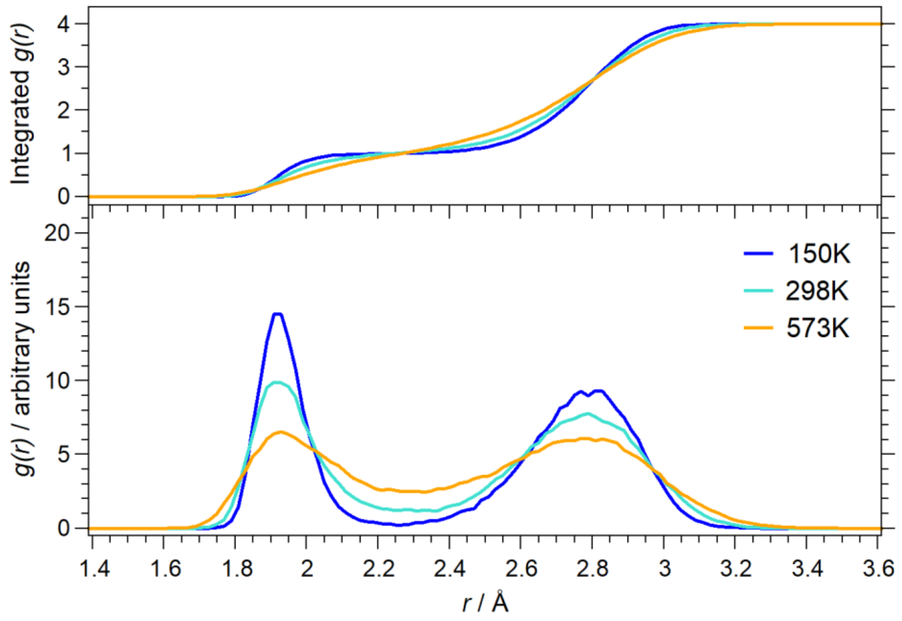

Figure S10b: F-Al RDFs of $AlPO_4$-AST_TMA

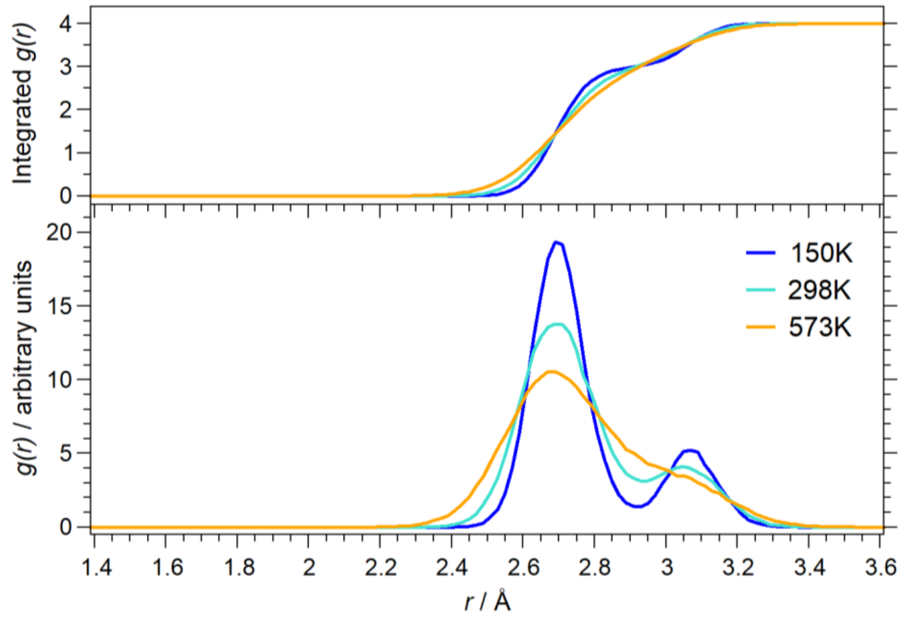

Figure S10c: F-P RDFs of $AlPO_4$-AST_TMA

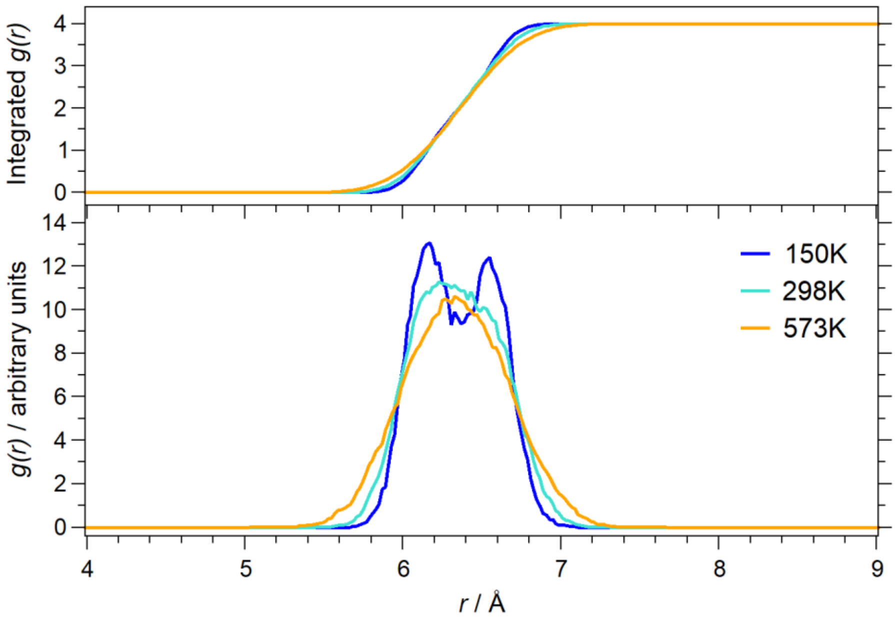

Figure S10d: F-N RDFs of $AlPO_4$-AST_TMA

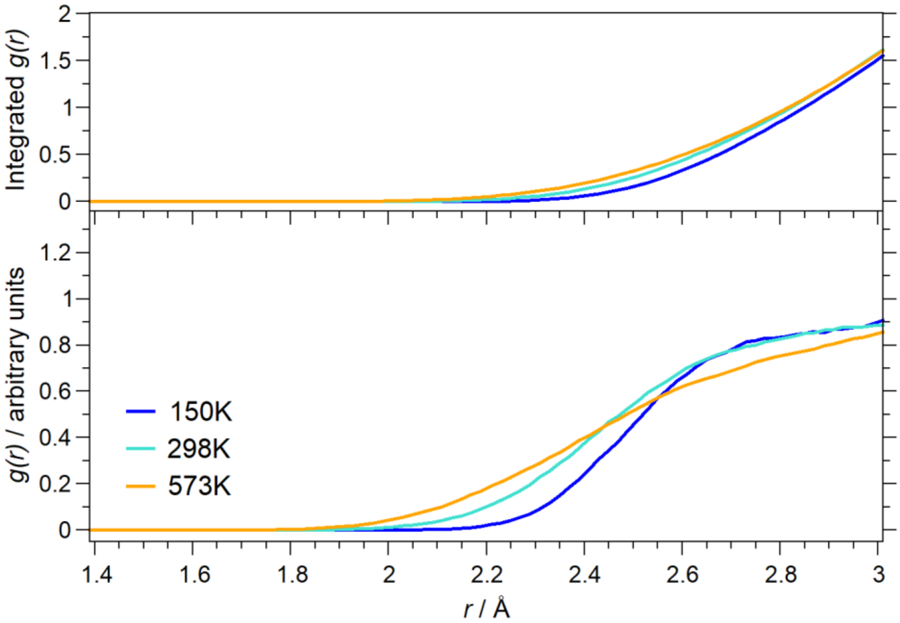

Figure S10e: H-O RDFs of $AlPO_4$-AST_TMA

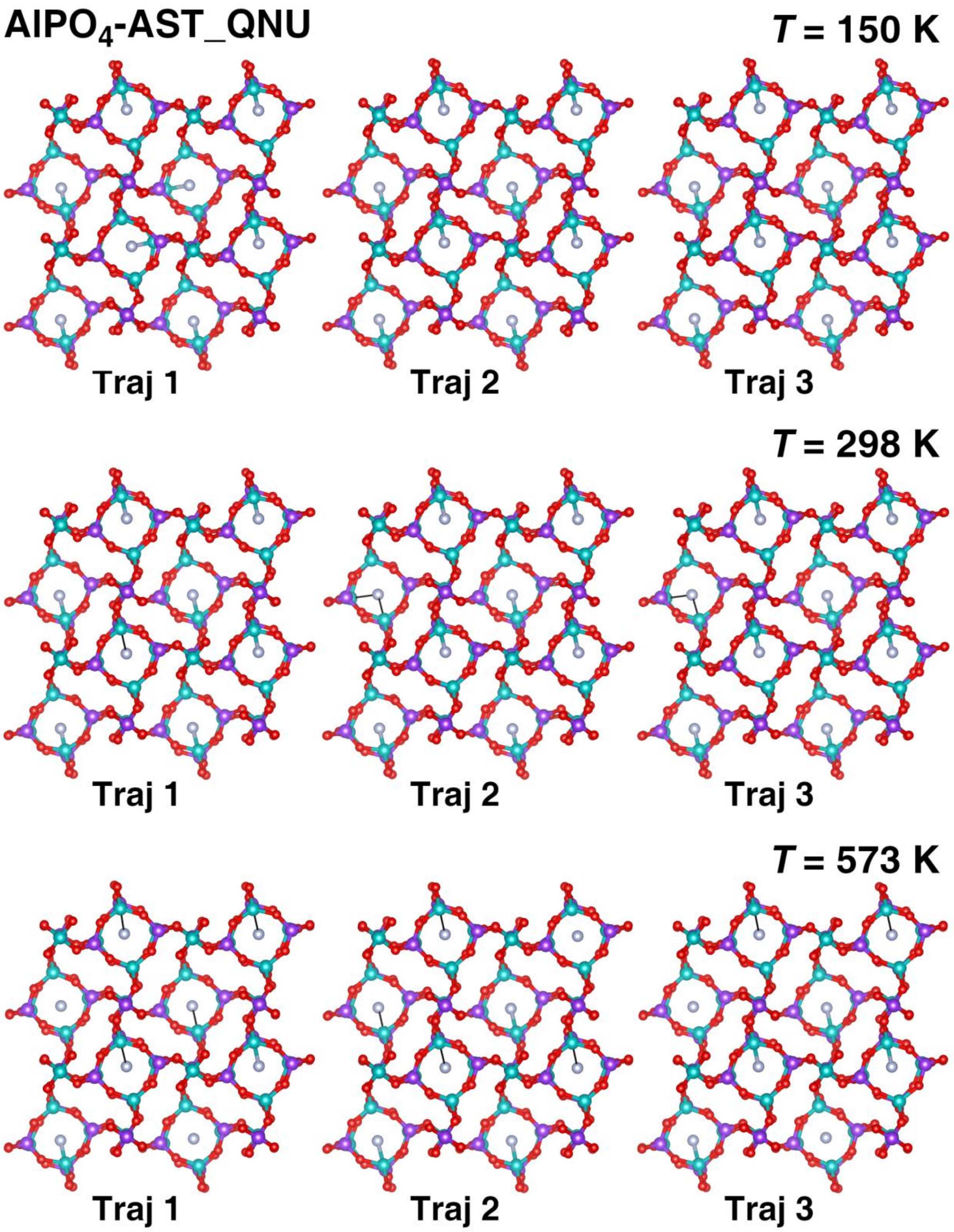

Figure S11a: Average structures of $AlPO_4$-AST_QNU obtained from independent AIMD runs (framework + fluoride anions).

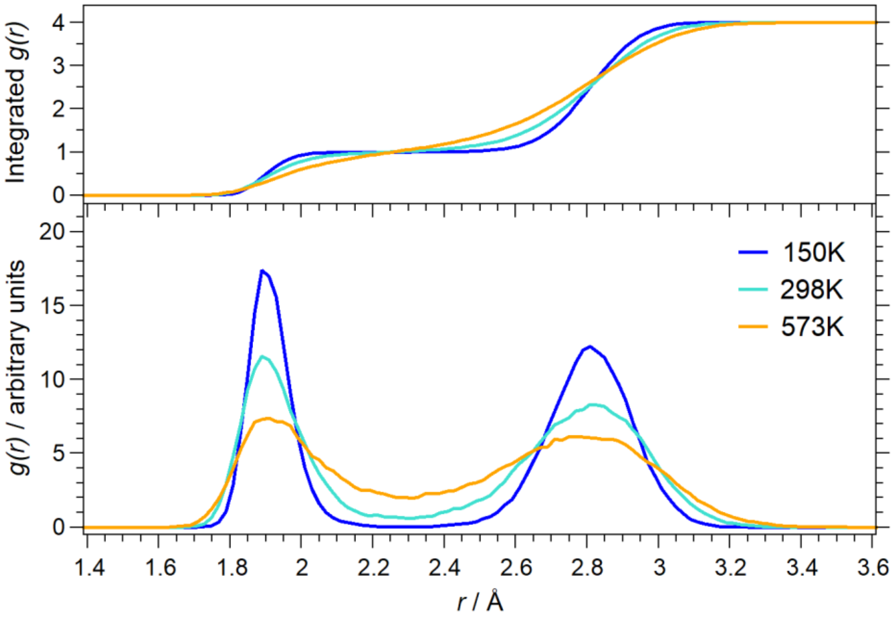

Figure S11b: F-Al RDFs of $AlPO_4$-AST_QNU

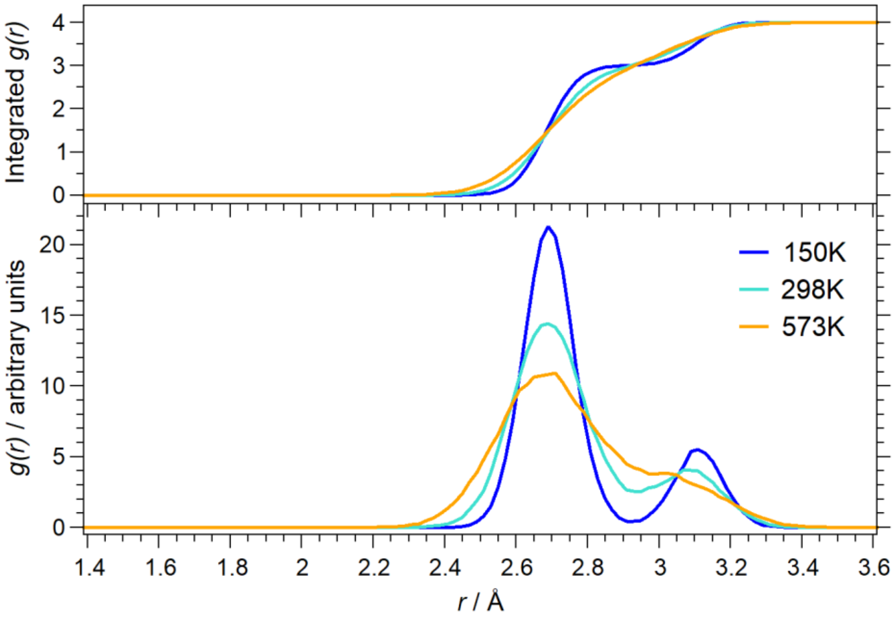

Figure S11c: F-P RDFs of $AlPO_4$-AST_QNU

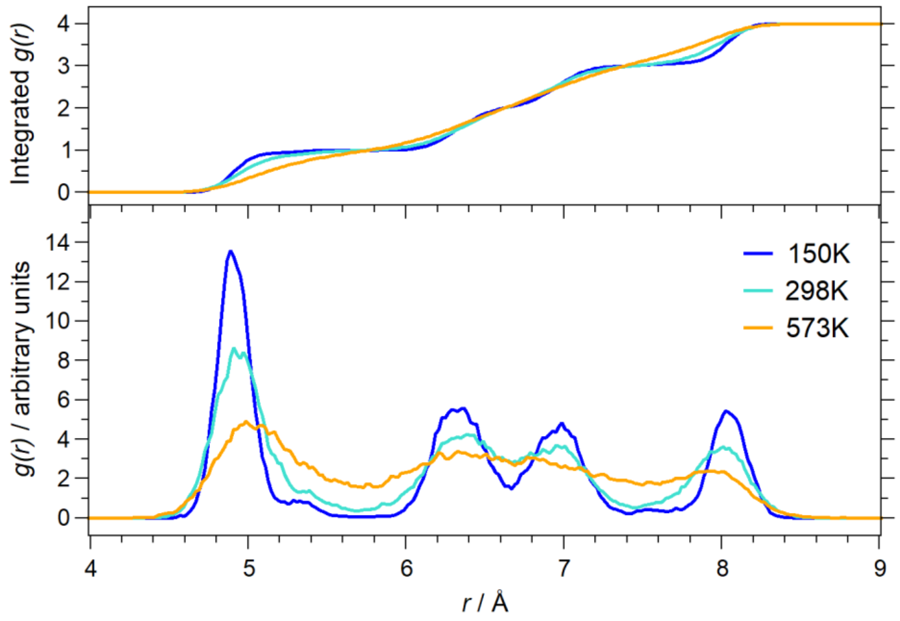

Figure S11d: F-N RDFs of $AlPO_4$-AST_QNU

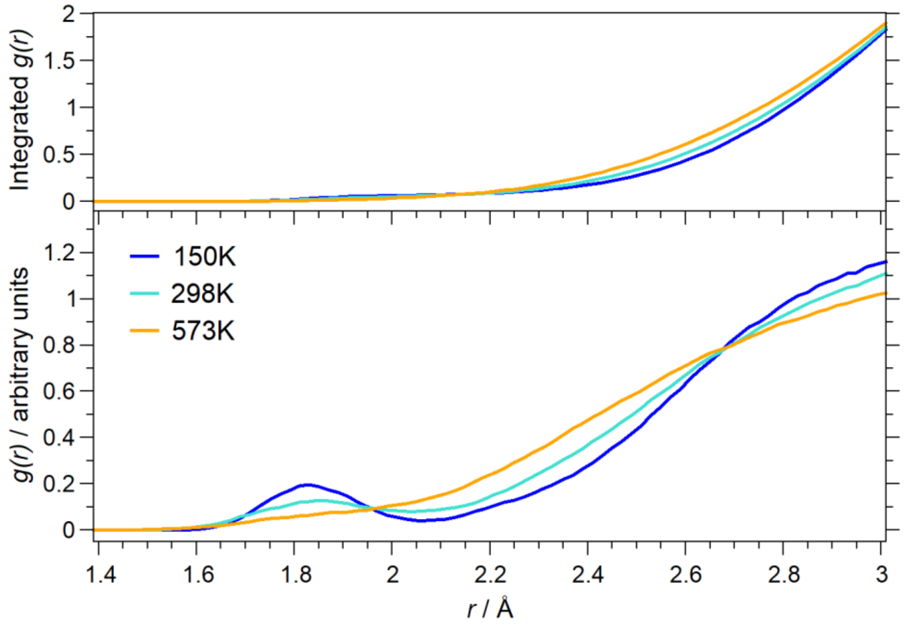

Figure S11e: H-O RDFs of $AlPO_4$-AST_QNU

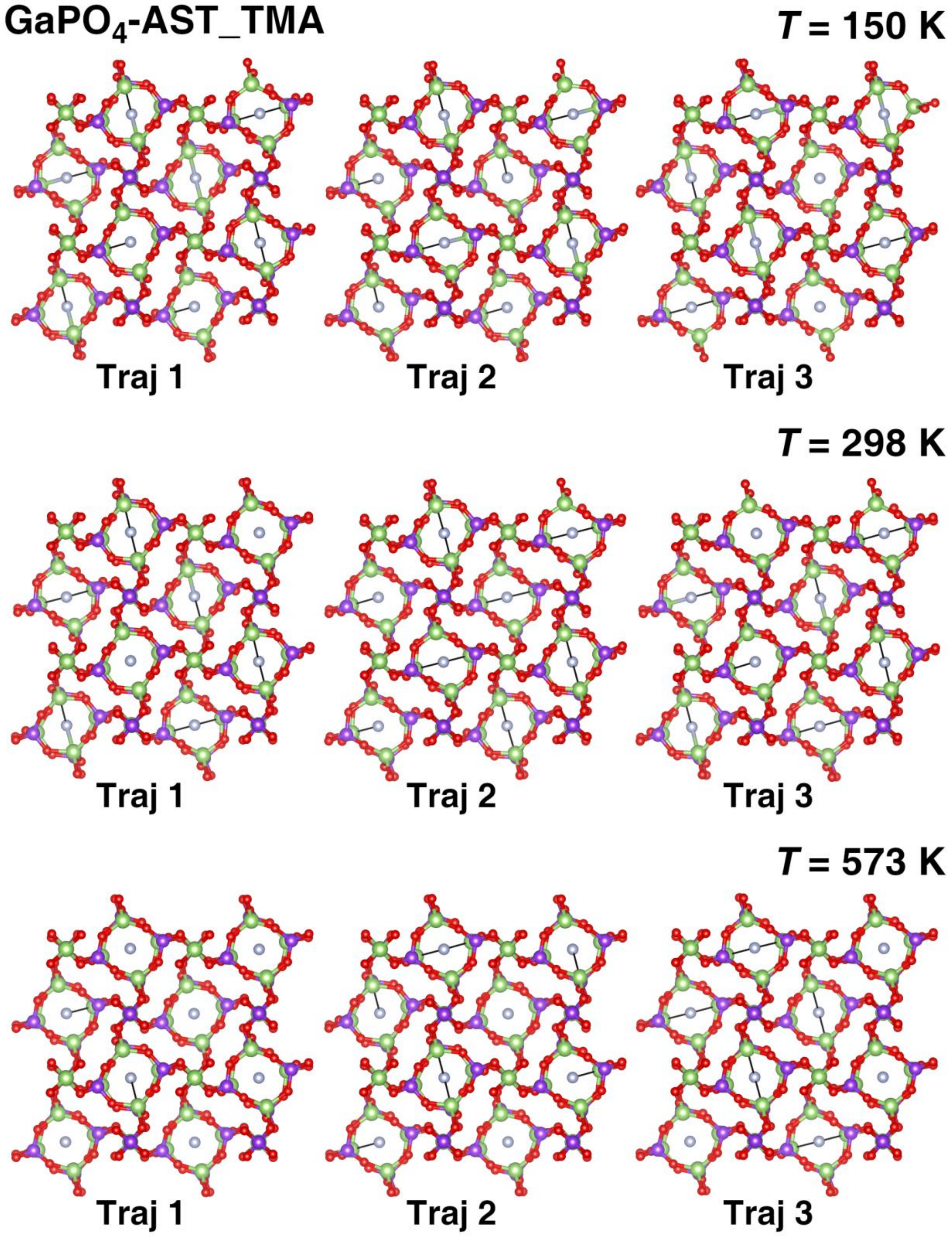

Figure S12a: Average structures of $GaPO_4$-AST_TMA obtained from independent AIMD runs (framework + fluoride anions).

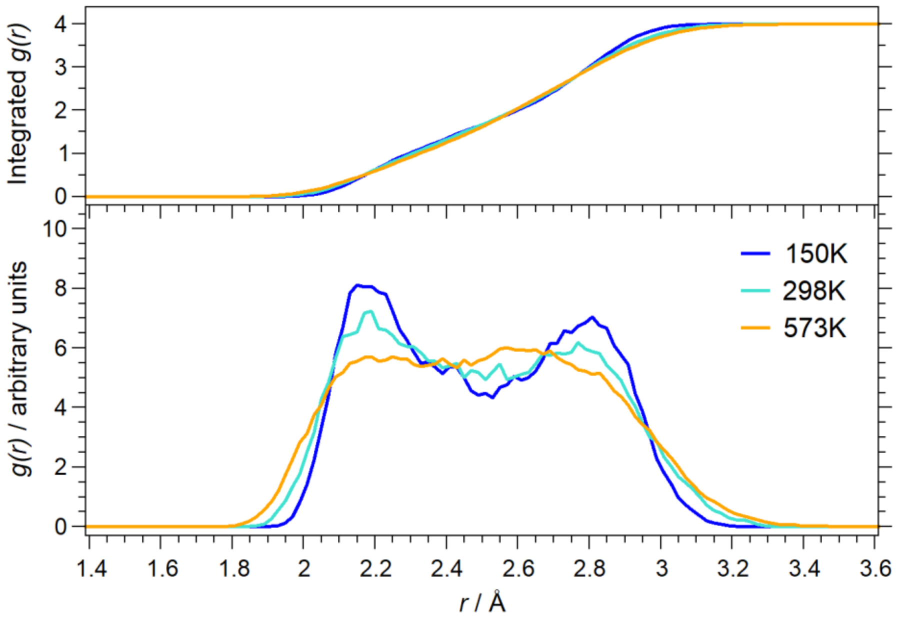

Figure S12b: F-Ga RDFs of $GaPO_4$-AST_TMA

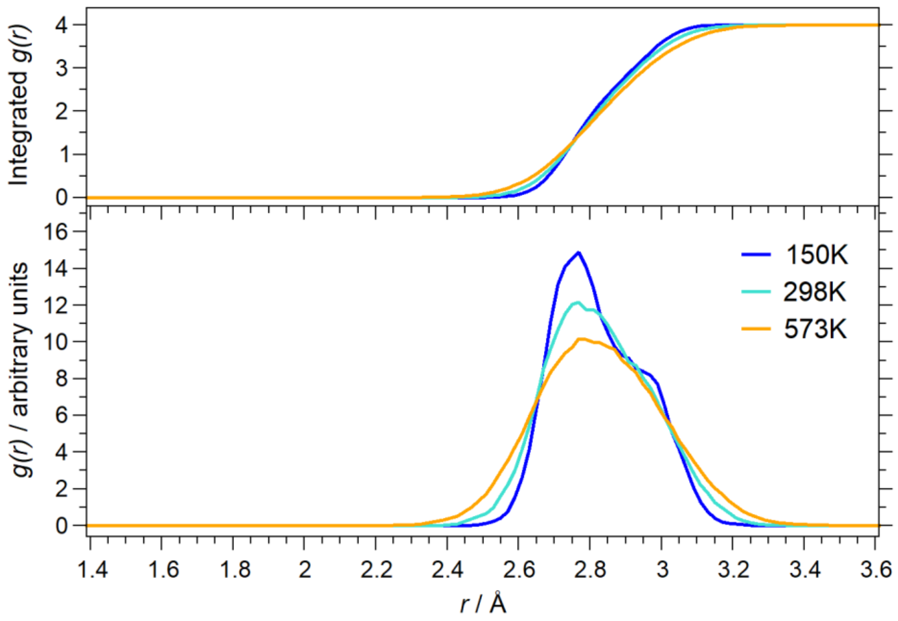

Figure S12c: F-P RDFs of $GaPO_4$-AST_TMA

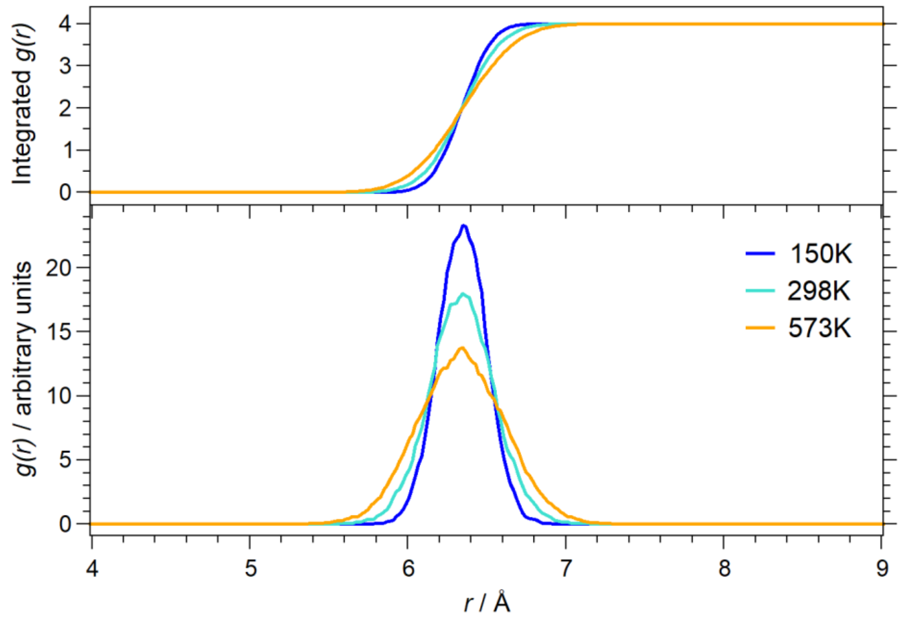

Figure S12d: F-N RDFs of $GaPO_4$-AST_TMA

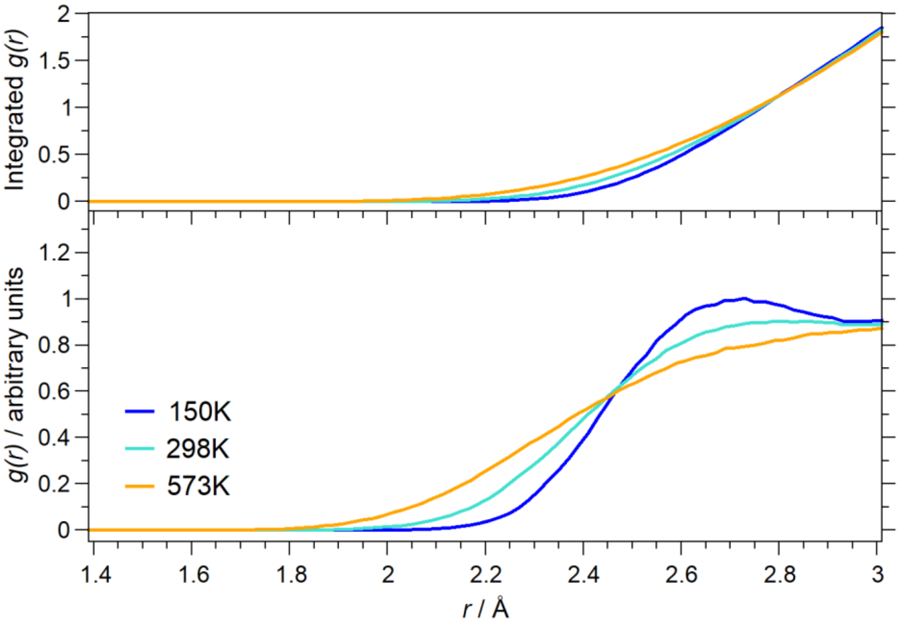

Figure S12e: H-O RDFs of $GaPO_4$-AST_TMA

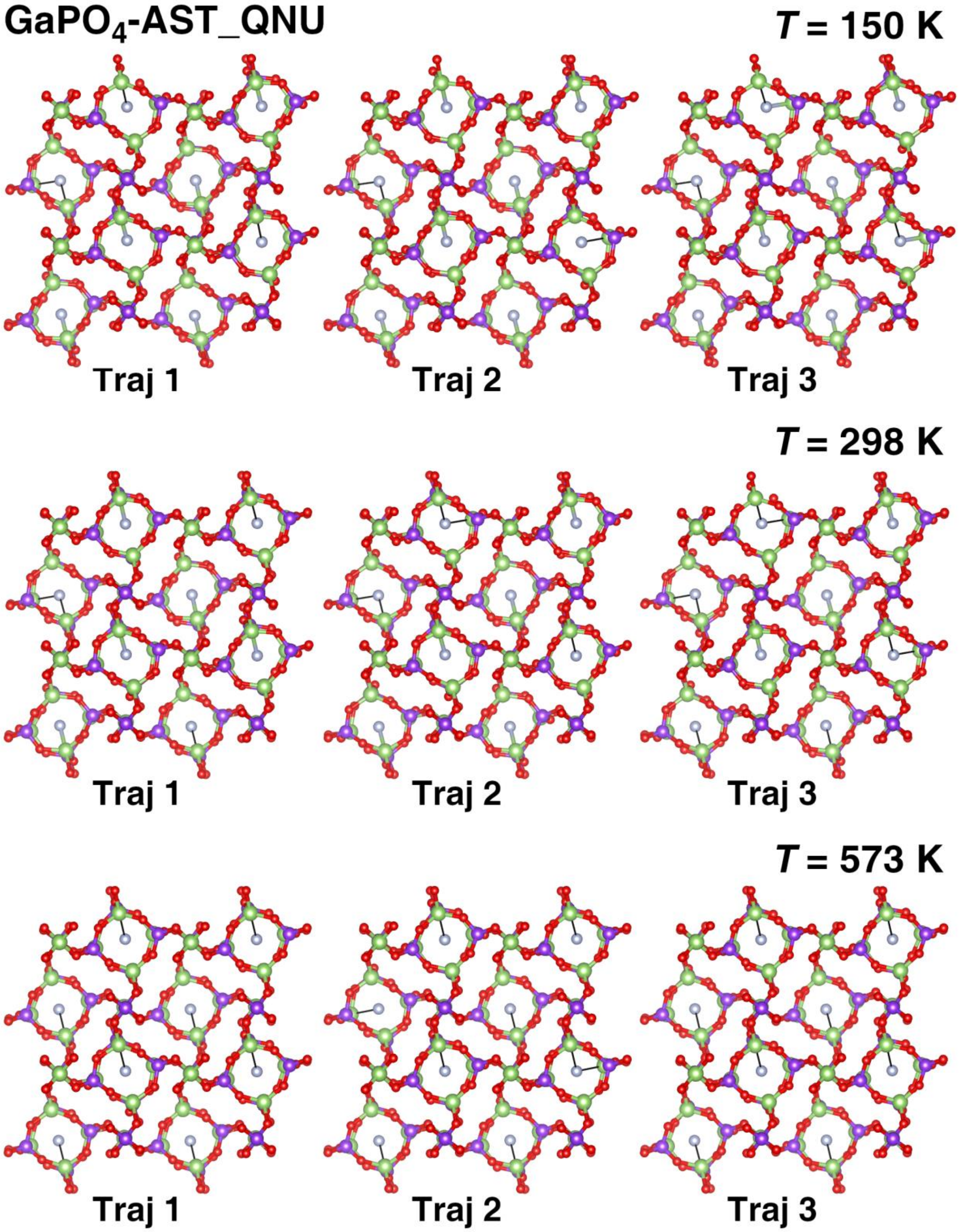

Figure S13a: Average structures of $GaPO_4$-AST_QNU obtained from independent AIMD runs (framework + fluoride anions).

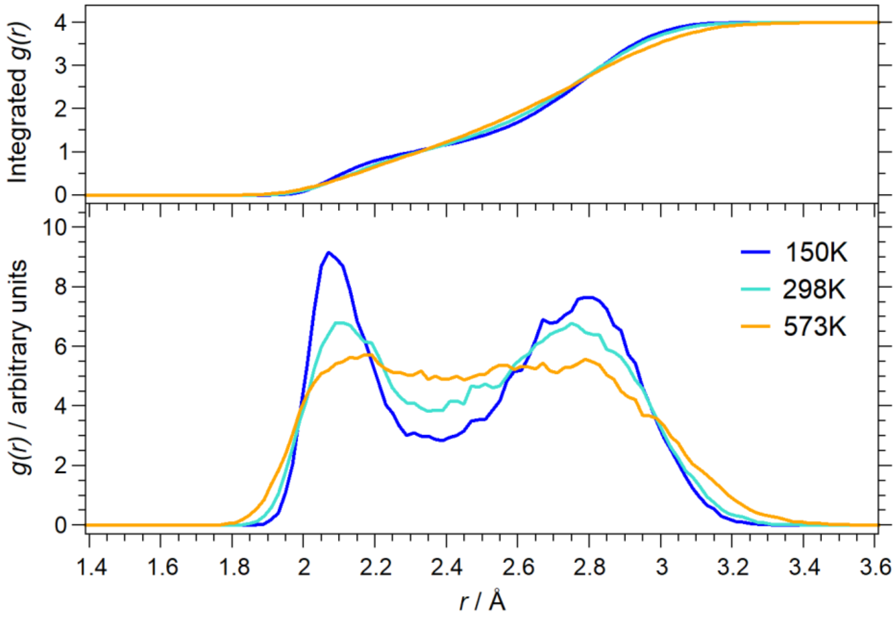

Figure S13b: F-Ga RDFs of $GaPO_4$-AST_QNU

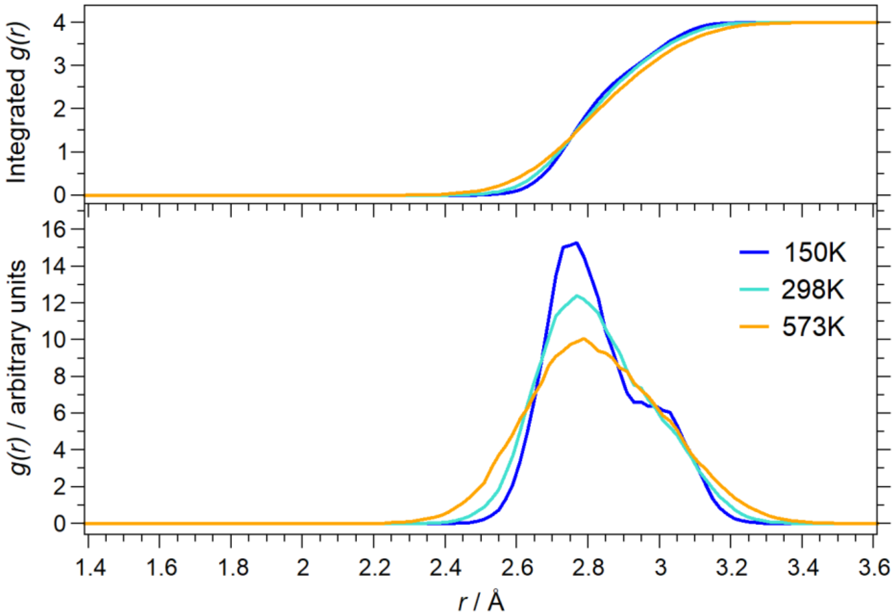

Figure S13c: F-P RDFs of $GaPO_4$-AST_QNU

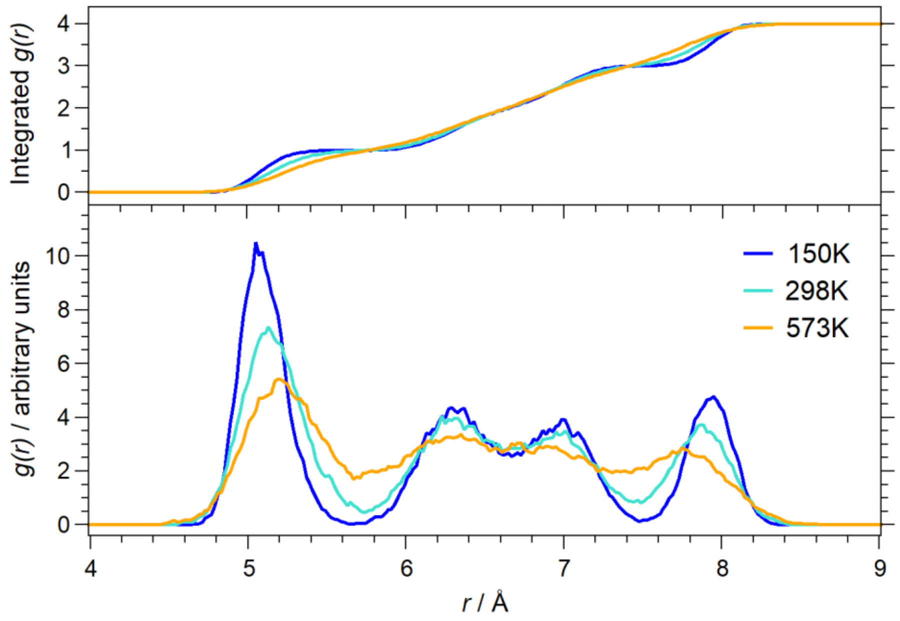

Figure S13d: F-N RDFs of $GaPO_4$-AST_QNU

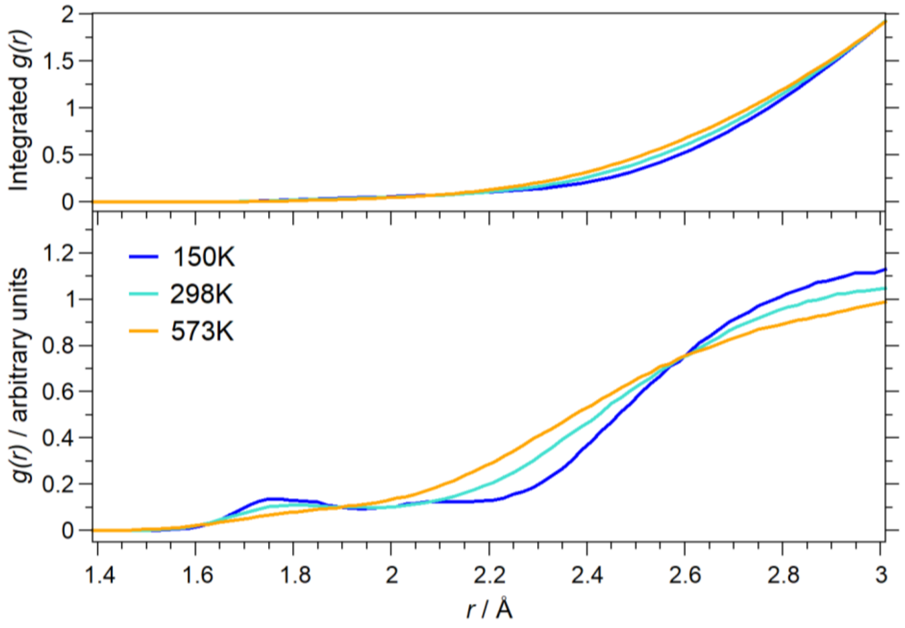

Figure S13e: H-O RDFs of $GaPO_4$-AST_QNU

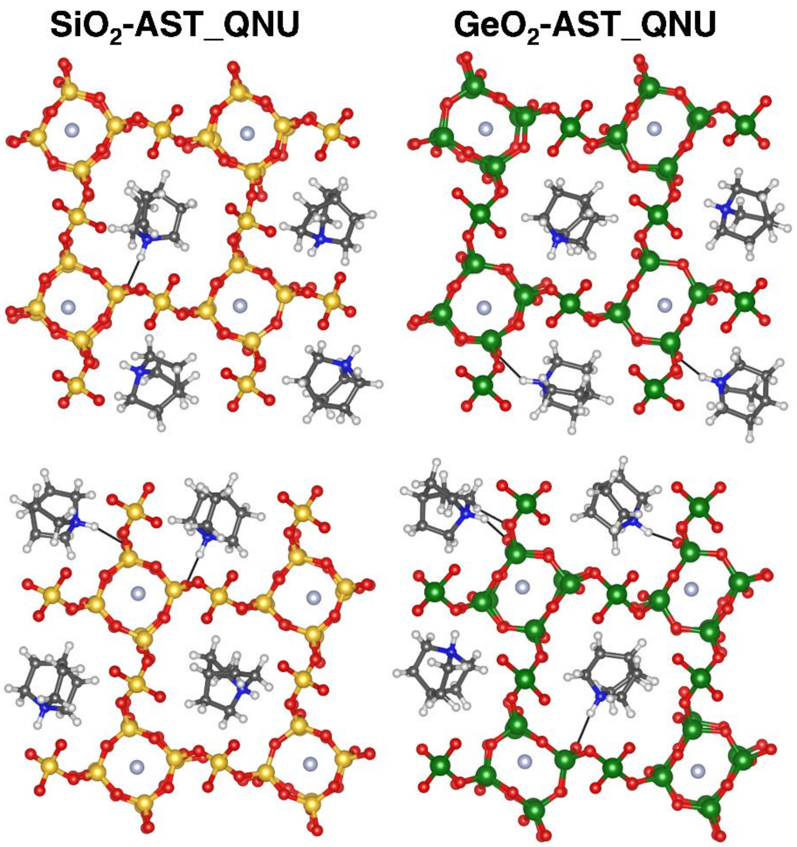

Figure S14: Representative last frames of AIMD trajectories obtained for T = 573 K for SiO_2-AST_QNU (left) and GeO_2-AST_QNU (right). Different sections perpendicular to c are shown separately (top: $0.25 < z < 0.75$; bottom: $-0.25 < z < 0.25$). H⋯O contacts up to 2.1 Å are shown as black lines.

SI4) Overview of other supplementary files

Further supplementary files are deposited in the **Figshare** repository: https://doi.org/10.6084/m9.figshare.12981557.v1

- `RMSDs_and_RDFs.xlsx`: Results from the analysis of AIMD trajectories (RMSDs, RDFs)
- `CP2K_sample_input_files.zip`: Sample input files for CP2K calculations (structure optimisations and AIMD calculations)
- `DFT-optimised_structures_CIFs.zip`: Results of the DFT structure optimisations (after symmetry search)
- `XXX-AST_AIMD_trajectories_PDB.zip`: AIMD trajectories (production stage only), XXX = $AlPO_4$/$GaPO_4$/GeO_2/SiO_2
- `AIMD_average_structures_CIFs.zip`: Average structures computed from AIMD trajectories (production stage only)
